# Gs-coupled GPCR signalling in AgRP neurons triggers sustained increase in food intake

**DOI:** 10.1038/ncomms10268

**Published:** 2016-01-08

**Authors:** Ken-ichiro Nakajima, Zhenzhong Cui, Chia Li, Jaroslawna Meister, Yinghong Cui, Ou Fu, Adam S. Smith, Shalini Jain, Bradford B. Lowell, Michael J. Krashes, Jürgen Wess

**Affiliations:** 1 Molecular Signaling Section, Laboratory of Bioorganic Chemistry, National Institute of Diabetes and Digestive and Kidney Diseases, Bethesda, Maryland 20892, USA; 2 Diabetes Endocrine and Obesity Branch, National Institute of Diabetes and Digestive and Kidney Diseases, Bethesda, Maryland 20892, USA; 3 Department of Applied Biological Chemistry, Graduate School of Agricultural and Life Sciences, The University of Tokyo, Bunkyo-ku, Tokyo 1138657, Japan; 4 Section on Neural Gene Expression, National Institute of Mental Health, Bethesda, Maryland 20892, USA; 5 Division of Endocrinology, Department of Medicine, Beth Israel Deaconess Medical Center, Harvard Medical School, Boston, Massachusetts 02115, USA

## Abstract

Agouti-related peptide (AgRP) neurons of the hypothalamus play a key role in regulating food intake and body weight, by releasing three different orexigenic molecules: AgRP; GABA; and neuropeptide Y. AgRP neurons express various G protein-coupled receptors (GPCRs) with different coupling properties, including G_s_-linked GPCRs. At present, the potential role of G_s_-coupled GPCRs in regulating the activity of AgRP neurons remains unknown. Here we show that the activation of G_s_-coupled receptors expressed by AgRP neurons leads to a robust and sustained increase in food intake. We also provide detailed mechanistic data linking the stimulation of this class of receptors to the observed feeding phenotype. Moreover, we show that this pathway is clearly distinct from other GPCR signalling cascades that are operative in AgRP neurons. Our data suggest that drugs able to inhibit this signalling pathway may become useful for the treatment of obesity.

## Abstract (web summary)

Hypothalamic Agouti-related peptide (AgRP) neurons play a key role in regulating food intake. Here, the authors report a novel pathway in which activation of Gs-coupled receptors on AgRP neurons leads to robust, sustained increase in food intake.

## Graphical abstract

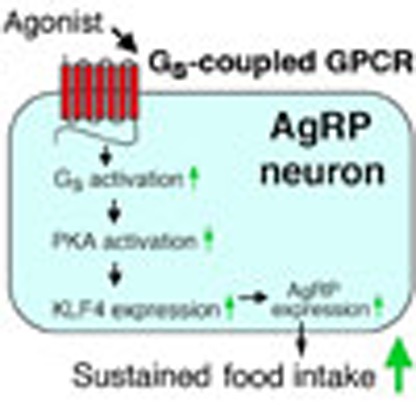

During the past decades, obesity has emerged as a major health crisis in most parts of the world^1^. Obesity represents a major risk factor for many severe diseases including type 2 diabetes, cancer and cardiovascular disease, thus posing an enormous socio-economic burden^2^.

Given the limited success of behavioural approaches towards achieving long-lasting weight loss, there is a clear need for effective pharmacological strategies to stem the current obesity epidemic^3^. Unfortunately, the number and efficacy of appetite-suppressing drugs that have been approved for clinical use is severely limited^3^.

To guide the design of novel appetite-suppressing drugs, it is essential to map the neuronal circuits that regulate food intake under physiological and pathophysiological conditions. In the present study, we focused on a small subpopulation of hypothalamic neurons located in the arcuate nucleus (ARC) of the hypothalamus, which synthesize and release agouti-related peptide (AgRP), a neuropeptide endowed with potent, long-lasting orexigenic activity^4^. These neurons, which are generally referred to as AgRP neurons, release two additional agents that promote acute feeding, neuropeptide Y (NPY) and GABA, a biogenic amine neurotransmitter^4^,^5^. At present, the relative roles of these three orexigenic agents in stimulating appetite in response to different hormones or neurotransmitters are not well understood.

Numerous studies have shown that AgRP neurons play a key role in regulating food intake and energy homeostasis^6^,^7^,^8^,^9^,^10^. For example, acute ablation of AgRP neurons in adult animals results in the cessation of feeding and, ultimately, starvation^6^,^7^.

As is the case with essentially all other cell types, the activity of AgRP neurons is predicted to be regulated by cell surface receptors belonging to the superfamily of G protein-coupled receptors (GPCRs), which are linked to distinct functional classes of G proteins^11^,^12^. So far, the functional consequences of activating the various GPCR/G protein signalling pathways in AgRP neurons have not been studied systematically. To address this issue, we started to employ a new set of pharmacological tools referred to as DREADDs (designer receptors exclusively activated by designer drugs). DREADDs represent mutant muscarinic acetylcholine receptors that are unable to bind acetylcholine or any other endogenous ligands^13^. However, DREADDs can be selectively activated by clozapine-N-oxide (CNO), a compound that is otherwise pharmacologically inert^13^,^14^,^15^,^16^,^17^. Importantly, we and others recently developed DREADDs endowed with distinct coupling G protein properties^15^,^16^,^17^, making it possible to monitor the *in vivo* consequences of activating distinct GPCR signalling pathways in a drug (CNO)-dependent fashion in specific cell types. Such studies are not possible to perform with native GPCRs, which, as a general rule, are expressed in multiple tissues and cell types^18^.

We recently generated mice that selectively express a G_q_-DREADD (hM3Dq; ref. 13) or a Gi-DREADD (hM4Di; ref. 13) in AgRP neurons of the ARC^19^. We found that CNO treatment of these animals resulted in greatly enhanced or reduced food intake, respectively^19^. In the present study, we selectively expressed a G_s_-linked DREADD (GsD; ref. 16) in mouse AgRP neurons. We demonstrate that drug-mediated stimulation of this receptor in AgRP neurons leads to a sustained increase in food intake that almost exclusively depends on the release of AgRP. In addition, we delineate a novel cellular pathway predicted to underlie this activity and show that this pathway is clearly distinct from the one involved in triggering acute food intake via activation of G_q_-coupled GPCRs in AgRP neurons. These new findings suggest that drugs capable of reducing G_s_ signalling in AgRP neurons might become clinically useful as appetite-suppressing drugs.

## Results

### Expression of a G_s_-coupled DREADD in AgRP neurons

In the present study, we selectively expressed a G_s_-coupled designer GPCR (G_s_ DREADD=GsD) in AgRP neurons of the mouse ARC. The generation and pharmacological properties of this construct have been described previously^14^. The GsD designer receptor, like other members of the DREADD family^13^,^15^,^16^,^17^, is no longer recognized by endogenous ligands, but can be selectively activated by CNO, an otherwise pharmacologically inert compound. To facilitate the detection of GsD expression in the mouse brain, we fused an enhanced green fluorescent protein (eGFP) tag to the C terminus of GsD (GsD-eGFP). Studies with transfected COS-7 cells demonstrated that the eGFP tag did not interfere with proper receptor expression and the ability of CNO to induce GsD-dependent G_s_ activation, as measured in cAMP accumulation assays (Supplementary Table 1).

**Figure 1:**
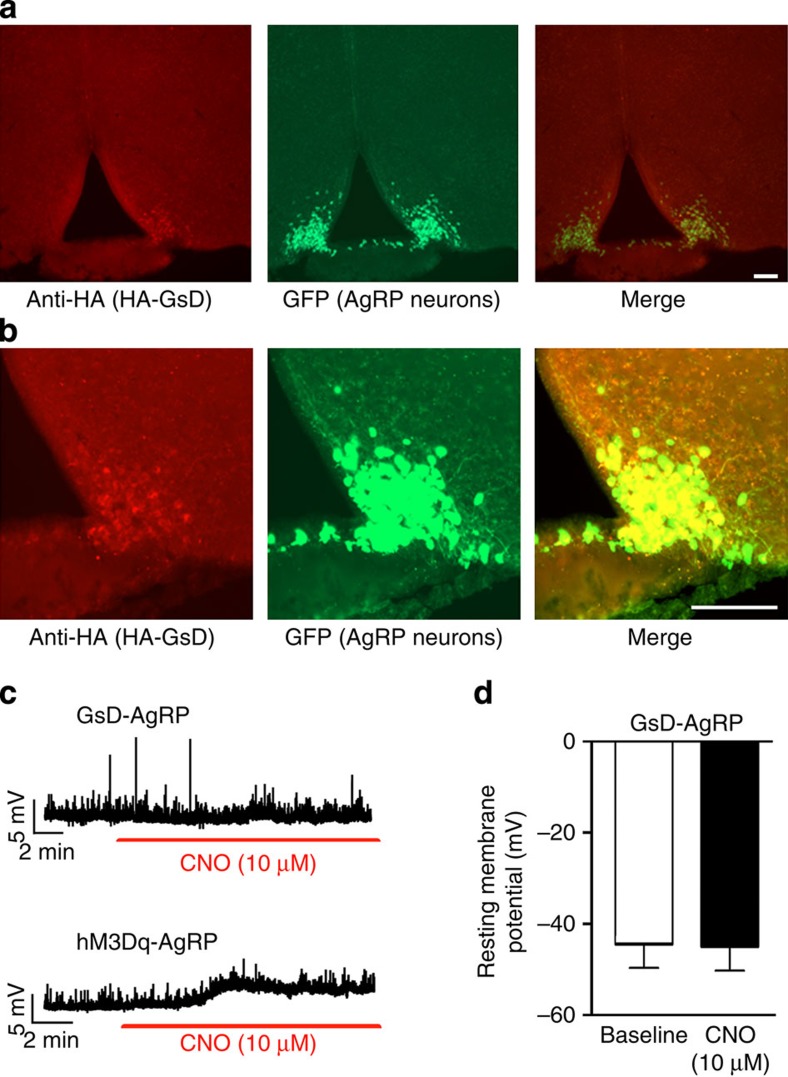
Expression of the GsD designer receptor in AgRP neurons of the ARC. (**a**,**b**) Specific expression of GsD in AgRP neurons of *Agrp-ires-Cre; R26-loxSTOPlox-L10-GFP* mice. In this mouse line, eGFP-L10 is selectively expressed, in a Cre-dependent fashion, in AgRP neurons^21^. Mice were injected unilaterally (right side) into the ARC with the AAV-hSyn-DIO-GsD-eGFP virus. The GsD designer receptor was detected by using an anti-HA tag antibody that recognizes the HA tag fused to the N terminus of GsD (left panels). In the centre panels, AgRP-positive neurons are visualized by green eGFP fluorescence (from *R26-L10-GFP*). The right panels represent a merged image indicating expression of GsD in AgRP neurons (AAV-injected side only). (**b**) Higher-magnification images. Scale bar, 100 μm. (**c**,**d**) CNO has no effect on GsD-AgRP neurons in whole-cell patch clamp electrophysiological recordings. (**c**) CNO has no significant effect on the membrane potential of GsD-AgRP neurons (GsD-AgRP neurons were identified by mCherry fluorescence in *AgRP-ires-Cre* mice injected with AAV-hSyn-DIO-rM3D(Gs)-mCherry virus). The upper trace shows a representative recording from a GsD-AgRP neuron in response to CNO bath application. For comparison, the lower trace depicts a representative recording derived from an hM3Dq-AgRP neuron, indicating that CNO depolarizes hM3Dq-AgRP neurons (also see ref. 19). In this latter experiment, *AgRP-ires-Cre* mice were injected with the AAV-hSyn-DIO-hM3Dq-mCherry virus, which codes for a G_q_-coupled DREADD (hM3Dq). Data are given as means±s.e.m. (*n*=7 neurons from 4–5 mice).

To selectively express GsD (GsD-eGFP) in mouse AgRP neurons, we used a Cre-recombinase-dependent adeno-associated virus (AAV) to target GsD to AgRP neurons. Stable transgene inversion was achieved by using FLEX Switch technology^20^. We stereotaxically injected an AAV that contained the GsD-eGFP coding sequence in the reverse orientation (AAV-hSyn-DIO-GsD-eGFP) into the ARC of *AgRP-ires-Cre* mice (bilaterally), which selectively express Cre recombinase in AgRP neurons. To demonstrate the selective expression of GsD-eGFP by AgRP neurons, we injected the same AAV into the ARC of *AgRP-ires-Cre; R26-loxSTOPlox-L10-GFP* mice (L10-GFP mice)^21^. In this mouse line, eGFP is selectively expressed, in a Cre-dependent fashion, in AgRP neurons^21^. GsD-eGFP expression was detected by an antibody directed against the eGFP tag (note that no native GFP fluorescence was observed with this construct, most likely due to low GsD-eGFP expression levels). This approach revealed the selective expression of the GsD-eGFP designer receptor in AgRP neurons of the ARC (Fig. 1a,b). For brevity, we refer to the AAV-hSyn-DIO-GsD-eGFP-injected *AgRP-ires-Cre* mice simply as ‘GsD-AgRP mice'.

For control purposes, we used the same approach to inject the AAV-hSyn-DIO-hM3Dq-mCherry virus into the ARC of *AgRP-ires-Cre* mice^19^ (note that hM3Dq represents a G_q_-coupled DREADD). We refer to these mice, which express the hM3Dq designer receptor selectively in AgRP neurons^19^, as ‘hM3Dq-AgRP mice' throughout the manuscript. Whole-cell patch clamp electrophysiological recordings revealed clear differences in CNO responses between GsD-AgRP and hM3Dq-AgRP neurons. We previously reported that CNO rapidly depolarizes the resting membrane potential of hM3Dq-AgRP neurons (see Fig. 1c, bottom panel, for a representative trace; see ref. 19). In contrast, bath application of CNO (10 μM) had no significant effect on the membrane potential of GsD-AgRP neurons (baseline, −44.3±5.5 mV; after CNO, −44.8±5.6 mV; *n*=7; Fig. 1c, top panel, d).

**Figure 2:**
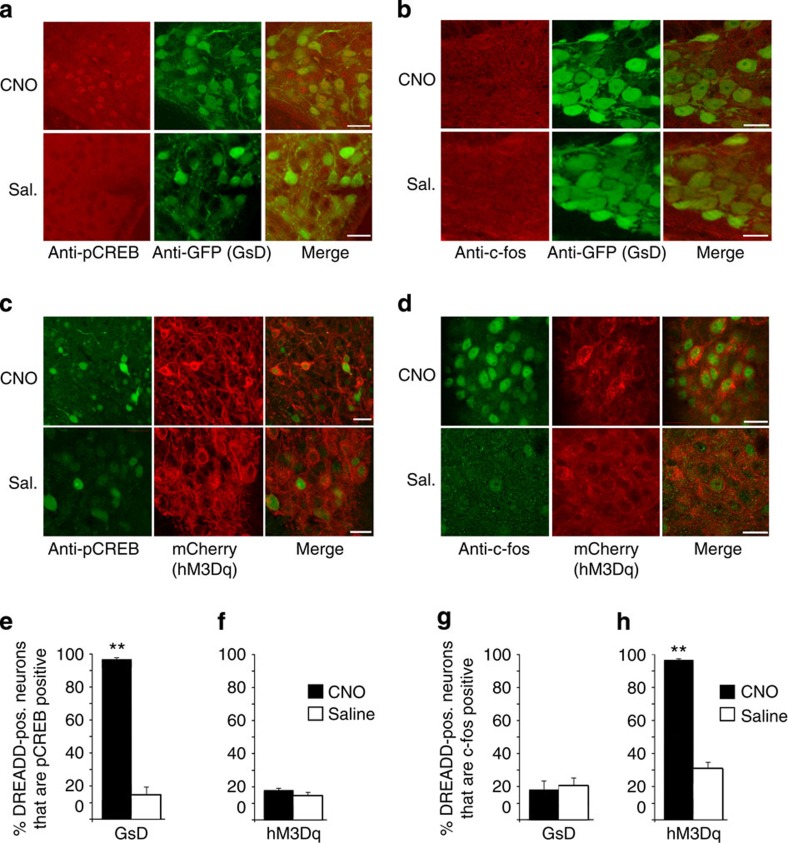
The GsD designer receptor selectively promotes pCREB expression in AgRP neurons. (**a**,**b**) AAV-mediated functional expression of GsD (GsD-eGFP) in AgRP neurons. pCREB (**a**) or c-fos (**b**) expression in AgRP neurons of GsD-AgRP mice was studied after CNO (1 mg kg^−1^) or saline (Sal.; control) treatment. Representative confocal images are shown. pCREB and c-fos expression were revealed by anti-pCREB and anti-c-fos antibodies, respectively (red staining). The GsD receptor was detected by using an anti-GFP antibody that recognized the eGFP tag fused to the C terminus of GsD (green staining). The images show selective induction of pCREB expression in AgRP neurons of CNO-treated GsD-AgRP mice. (**c**,**d**) AAV-mediated functional expression of hM3Dq (hM3Dq-mCherry) in AgRP neurons. pCREB (**c**) or c-fos (**d**) expression in AgRP neurons of hM3Dq-AgRP mice was studied after CNO (1 mg kg^−1^) or saline (control) treatment. Representative confocal images are shown. pCREB and c-fos expression were revealed by anti-pCREB and anti-c-fos antibodies, respectively (green staining). The red staining (mCherry fluorescence) visualizes hM3Dq-expressing neurons. The images show selective induction of c-fos expression in AgRP neurons of CNO-treated hM3Dq-AgRP mice. Scale bar, 20 μm. (**e**–**h**) Quantification of confocal images shown in **a**–**d**. The percentage of DREADD-positive (pos.) neurons (as assessed by GFP staining or mCherry fluorescence) staining positive for pCREB or c-fos was calculated. Data are expressed as means±s.e.m. (12–16 sections from three different mice per group were analysed). **P<0.01 (Student's *t*-test).

To trigger GsD signalling selectively in mouse AgRP neurons, we injected GsD-AgRP mice with a single dose of CNO (1 mg kg^−1^ intraperitoneal (i.p.)) or saline (as a negative control). Gene expression studies were performed 2 h after CNO injections. CNO treatment of GsD-AgRP mice had no detectable effect on c-fos expression in AgRP neurons (Fig. 2b,g and Supplementary Fig. 1b). GsD activation is predicted to increase cellular cAMP levels and to stimulate the activity of protein kinase A (PKA)^14^,^22^, resulting in the phosphorylation of many cellular proteins including the transcription factor CREB. Consistent with this concept, we found that CNO treatment of GsD-AgRP mice promoted CREB phosphorylation selectively in AgRP neurons (Fig. 2a,e and Supplementary Fig. 1a). In contrast, saline-injected GsD-AgRP mice showed little or no pCREB staining in AgRP neurons (Fig. 2a,e and Supplementary Fig. 1a). These data clearly indicate that the GsD designer receptor is functional in AgRP neurons.

Interestingly, hM3Dq-AgRP mice displayed a functional profile that was opposite to that observed with the GsD-AgRP mice. In hM3Dq-AgRP mice, CNO treatment triggered strong c-fos staining in hM3Dq-expressing AgRP neurons, while little or no c-fos expression was observed with saline-treated hM3Dq-AgRP mice (Fig. 2d,h and Supplementary Fig. 1d; also see ref. 19). In contrast to GsD-AgRP mice, CNO treatment of hM3Dq-AgRP mice did not result in a detectable increase in CREB phosphorylation in AgRP neurons (Fig. 2c,f and Supplementary Fig. 1c). A quantitative summary of the pCREB and c-fos expression data is given in Fig. 2e–h.

### GsD activation promotes sustained feeding

**Figure 3:**
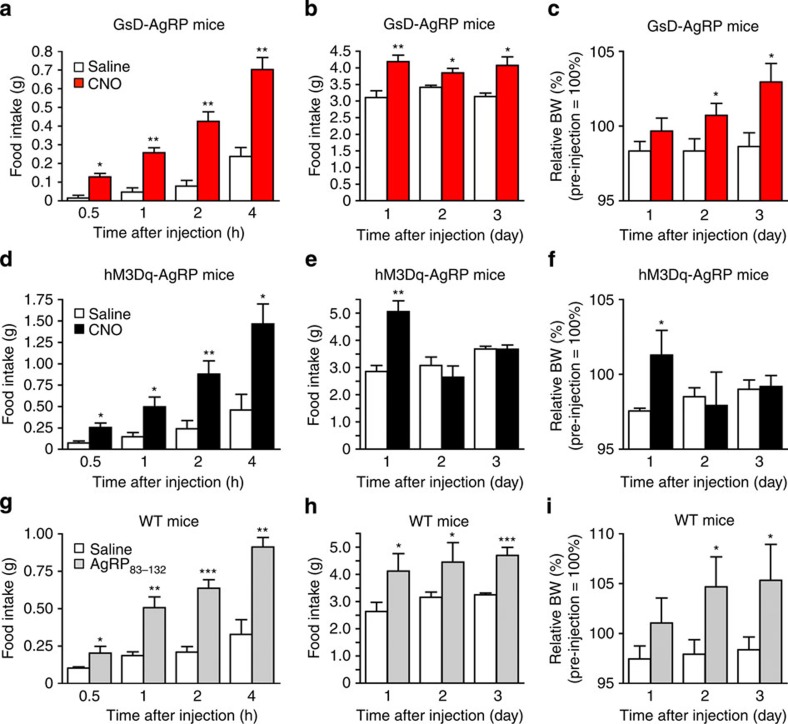
Food intake studies with GsD-AgRP, hM3Dq-AgRP and WT control mice. CNO-mediated activation of GsD in AgRP neurons promotes food intake both acutely (**a**) and over a 3-day period (**b**), and increases body weight (BW) (**c**). Measurements were carried after a single i.c.v. injection of either saline or CNO (1 μg) into GsD-AgRP mice. CNO-induced activation of hM3Dq in AgRP neurons stimulates food intake acutely (**d**) but not chronically (**e**). BW was increased only on day 1 (**f**). Measurements were carried after a single i.c.v. injection of either saline or CNO (1 μg) into hM3Dq-AgRP mice. (**g**–**i**) Treatment of WT control mice with an active AgRP fragment (AgRP_83–132_) promotes food intake acutely (**g**) and over a 3-day period (**h**), and leads to increased BW (**i**). Measurements were carried out after a single i.c.v. injection of either saline or AgRP_83–132_ (3 μg). Injections were carried out between 9:00 and 10:00. All experiments were carried out with 12- to 16-week-old male mice. Data are given as means±s.e.m. (*n*=4–7 mice per group). *P<0.05, **P<0.01, ***P<0.001, as compared with the corresponding saline-injected group (Student's *t*-test).

We next examined whether GsD signalling in AgRP neurons was able to modulate food intake. Specifically, we injected GsD-AgRP mice with a single dose of CNO (1 μg intracerebroventricular (i.c.v.)) or saline early in the morning (between 9:00 and 10:00) when mice are satiated and consume little food. In most feeding experiments, we delivered CNO i.c.v. since this route of application yielded more consistent data, as compared with i.p. administration of CNO. Subsequently, we monitored acute food intake over a 4-h period as well as long-lasting effects on food intake over the following 3 days. In wild-type (WT) control mice, CNO administration (1 μg i.c.v.) had no significant effect on food intake (Supplementary Fig. 2). In contrast, CNO treatment of GsD-AgRP mice triggered a robust increase in food intake during the first 4 h after CNO injection (Fig. 3a). Surprisingly, this CNO-induced orexigenic effect was very long-lasting (for 3 days; Fig. 3b). The orexigenic effects of CNO were dose dependent, as shown in Supplementary Fig. 3. Consistent with the food intake data, CNO-injected GsD-AgRP mice displayed a significant increase in body weight on days 2 and 3 after CNO administration (Fig. 3c).

For control purposes, we carried out analogous experiments with hM3Dq-AgRP mice. As reported previously^19^, CNO treatment (1 μg i.c.v.) of hM3Dq-AgRP mice triggered a striking increase in food intake during the first 24 h after CNO administration (Fig. 3d,e). This effect was even more robust than that observed with GsD-AgRP mice (please note that the y axes have different scales in Fig. 3a,d). However, in contrast to GsD-AgRP mice in which CNO triggered long-lasting orexigenic effects (Fig. 3b), CNO-treated hM3Dq-AgRP mice showed normal food intake on days 2 and 3 after CNO injection (Fig. 3e). In agreement with this observation, CNO-treated hM3Dq-AgRP mice did not show any changes in body weight on days 2 and 3 after CNO injection, as compared with saline-treated hM3Dq-AgRP mice (Fig. 3f).

Previous studies have demonstrated that AgRP treatment of rodents has long-lasting effects on food intake^23^,^24^. We therefore speculated that the sustained feeding phenotype displayed by the CNO-injected GsD-AgRP mice was due to GsD-dependent AgRP release. To address this issue, we injected WT control mice with a single dose (3 μg i.c.v.) of an active AgRP fragment, AgRP_83–132_, using the same experimental set-up as described above. Strikingly, the AgRP_83–132_-injected WT mice showed increases in food intake and body weight that were characterized by a pattern very similar to that observed with CNO-treated GsD-AgRP mice (Fig. 3g–i).

### GsD-induced feeding requires AgRP release

To further test the hypothesis that the orexigenic effects observed after activation of the GsD designer receptor require AgRP, we used two different experimental strategies.

**Figure 4:**
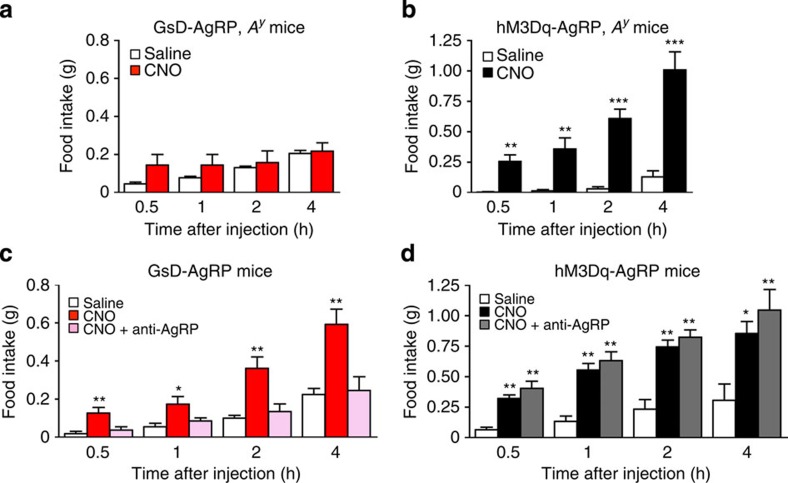
AgRP release is essential for the orexigenic effects observed with GsD-AgRP but not with hM3Dq-AgRP mice. (**a**,**c**) The orexigenic effect triggered by GsD activation in AgRP neurons is abolished in the presence of the *A*^*y*^ mutation (**a**) or by treatment with an anti-AgRP antibody (**c**). (**b**,**d**) The orexigenic effect caused by activation of hM3Dq signalling in AgRP neurons remains unaffected in the presence of the *A*^*y*^ mutation (**b**) or by treatment with an anti-AgRP antibody (**d**). Mice received a single i.c.v. injection of either saline, CNO (0.5 μg) or CNO (0.5 μg) plus an anti-AgRP antibody (1 μg). All experiments were carried out with 10- to 16-week-old male mice. Data are given as means±s.e.m. (*n*=4–8 mice per group). *P<0.05, **P<0.01, ***P<0.001, as compared with the corresponding saline-injected group (**a**,**b**, Student's *t*-test; **c**,**d**, one-way analysis of variance followed by Dunnett's *post hoc* test).

First, we generated GsD-AgRP mice that carried a single copy of the *agouti* ‘lethal yellow' mutant allele (*A*^*y*^) (GsD-AgRP, *A*^*y*^ mice). For comparison, we also introduced the *A*^*y*^ mutation into hM3Dq-AgRP mice (hM3Dq-AgRP, *A*^*y*^ mice). The *A*^*y*^ mutation leads to the ectopic expression of agouti protein in the brain (as well as other tissues) where it blocks central melanocortin receptors, in a fashion similar to AgRP^25^. As a result, mice carrying the *A*^*y*^ mutation are predicted to show reduced sensitivity to the orexigenic actions of AgRP^26^. We found that CNO failed to stimulate food intake in GsD-AgRP mice carrying the *A*^*y*^ mutation (Fig. 4a). In contrast, the presence of the *A*^*y*^ mutation did not interfere with the ability of CNO to promote robust food intake in hM3Dq-AgRP mice (Fig. 4b), in agreement with the outcome of a previous study employing optogenetic interrogation of AgRP neurons^26^.

Second, we co-injected GsD-AgRP mice with CNO (0.5 μg i.c.v.) and an anti-AgRP antibody (1 μg i.c.v.)^27^. Treatment of GsD-AgRP mice with the anti-AgRP antibody alone had no significant effect on food intake (Supplementary Fig. 4). Strikingly, the anti-AgRP antibody completely blocked the ability of CNO to promote food intake in GsD-AgRP mice (Fig. 4c). In contrast, treatment of hM3Dq-AgRP mice with the anti-AgRP antibody had no significant effect on CNO-mediated orexigenic effects (Fig. 4d).

These data clearly indicate that the orexigenic effects mediated by G_s_- and G_q_-coupled receptors selectively expressed in AgRP neurons involve different cellular/neuronal mechanisms.

### GsD-induced feeding does not require NPY/GABA release

A recent study^5^ demonstrated that the acute orexigenic effect observed with CNO-treated hM3Dq-AgRP mice requires the release of NPY and/or GABA. We therefore explored the possibility that NPY or GABA might also contribute to the CNO-mediated increase in food intake in GsD-AgRP mice. Initially, we demonstrated that treatment of GsD-AgRP mice with NPY (0.85 μg i.c.v.) caused a marked increase in food intake (Supplementary Fig. 5a), consistent with the well-known orexigenic activity of NPY^28^. Co-injection of NPY with BIBO3304 (3 μg i.c.v.), a selective NPY Y_1_ receptor antagonist^29^, completely abolished NPY-induced feeding in GsD-AgRP mice (Supplementary Fig. 5a), indicating that the orexigenic effects of NPY are mediated by NPY Y_1_ receptors. Treatment of control mice (*AgRP-ires-Cre* mice that had been injected with AAV-hSyn-DIO-mCherry) with BIBO3304 alone had no effect on basal food intake (Supplementary Fig. 5b).

**Figure 5:**
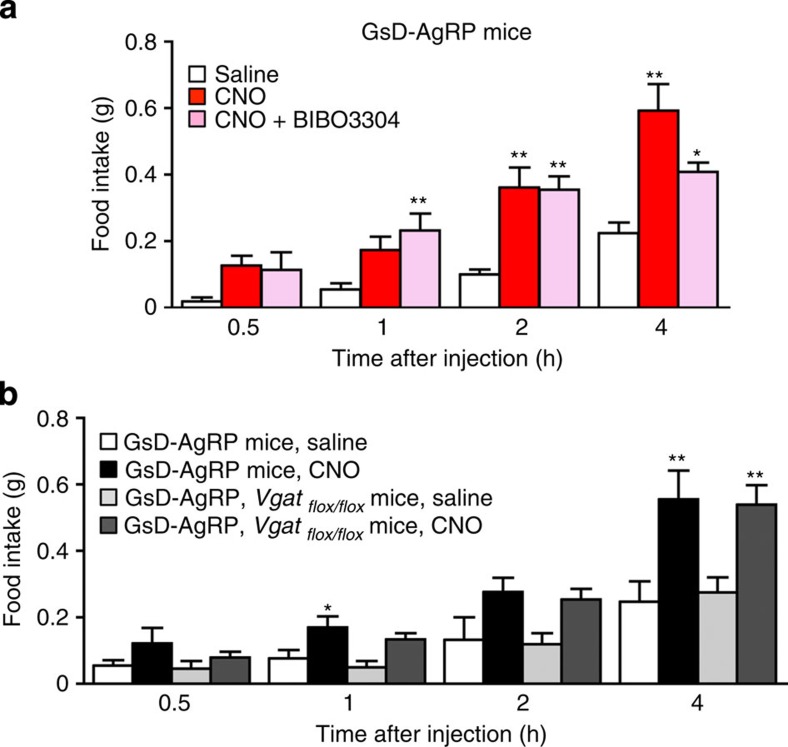
CNO-induced stimulation of food intake in GsD-AgRP mice does not require NPY or GABA release. (**a**) Treatment of GsD-AgRP mice with BIBO3304, a selective NPY Y_1_ receptor antagonist, has little or no effect on CNO-induced increases in food intake. Mice received a single i.c.v. injection of either saline, CNO (0.5 μg) or CNO (0.5 μg) plus BIBO3304 (3 μg). (**b**) The orexigenic effects following GsD activation in AgRP neurons do not require GABA release from AgRP neurons. GsD-AgRP mice or GsD-AgRP mice lacking *Vgat* selectively in AgRP neurons (GsD-AgRP*-ires-Cre, Vgat*_*flox/flox*_ mice) received a single i.p. injection of either saline or CNO (1 mg kg^−1^). All experiments were carried out with 11- to 17-week-old male mice. Data are given as means±s.e.m. (*n*=4–8 mice per group).*P<0.05, **P<0.01, as compared with the corresponding saline-injected group (one-way analysis of variance followed by Dunnett's *post hoc* test).

We next injected GsD-AgRP mice with either CNO alone (0.5 μg i.c.v.) or with CNO plus BIBO3304 (0.5 and 3 μg i.c.v., respectively). BIBO3304 treatment had no significant effect on CNO-induced food intake at the 0.5, 1 and 2 h time points but caused a partial reduction in food intake 4 h after CNO injection (Fig. 5a). This observation suggests that NPY does not make a major contribution to the orexigenic effect caused by activation of the GsD designer receptor in AgRP neurons.

To test the possibility that GABA release from AgRP neurons might contribute to the ability of CNO to increase food intake in GsD-AgRP mice, we generated GsD-AgRP mice that selectively lacked the vesicular GABA transporter (*Vgat*) in AgRP neurons (GsD-AgRP*-ires-Cre, Vgat*_*flox/flox*_ mice). In these mice, GABA cannot be released from AgRP neurons since it cannot be packaged in synaptic vesicles. We found that selective deletion of the *Vgat* gene in AgRP neurons had no significant effect on the ability of CNO (1 mg kg^−1^ i.p.) to stimulate food intake in GsD-AgRP mice (Fig. 5b). This observation indicates that the orexigenic effect triggered by activation of the GsD designer receptor in AgRP neurons does not require the release of GABA.

### GsD activation promotes hypothalamic AgRP release

**Figure 6:**
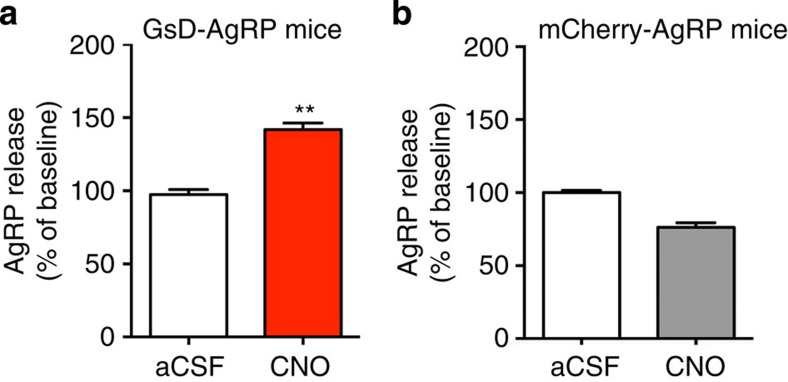
Activation of GsD expressed by AgRP neurons promotes hypothalamic AgRP release. (**a**) CNO (15 μM) treatment of hypothalamic slices prepared from GsD-AgRP mice triggers a significant increase in AgRP release (incubation period: 1 h). CNO-induced AgRP release was normalized to basal AgRP release determined during an initial 1-h incubation period in the presence of medium (aCSF) alone. (**b**) This stimulatory CNO effect was not observed with hypothalamic slices prepared from mCherry-AgRP control mice. All experiments were carried out with 10- to 16-week-old mice. Data are given as means±s.e.m. (*n*=11–13 mice per group; **P<0.01; Student's *t*-test).

To further confirm that activation of GsD expressed by AgRP neurons promotes hypothalamic AgRP release, we carried out *ex vivo* AgRP secretion experiments. Specifically, we incubated hypothalamic slices prepared from GsD-AgRP mice, with artificial cerebrospinal fluid (aCSF) for 1 h at 37 °C (to determine basal AgRP release), followed by a second 1-h incubation in the presence of CNO (15 μM). In parallel, we carried out analogous experiments with hypothalamic slices prepared from mCherry-AgRP control mice (*AgRP-ires-Cre* mice that had been injected bilaterally with the AAV-hSyn-DIO-mCherry virus). Strikingly, CNO triggered a significant increase in AgRP release from hypothalamic preparations of GsD-AgRP mice (Fig. 6a). This effect was absent in hypothalamic slices prepared from mCherry-AgRP control mice (Fig. 6b). Taken together, these data clearly indicate that activation of GsD, a G_s_-coupled designer receptor, in AgRP neurons stimulates hypothalamic AgRP release.

### GsD activation increases *AgRP* and *KLF4* expression

**Figure 7:**
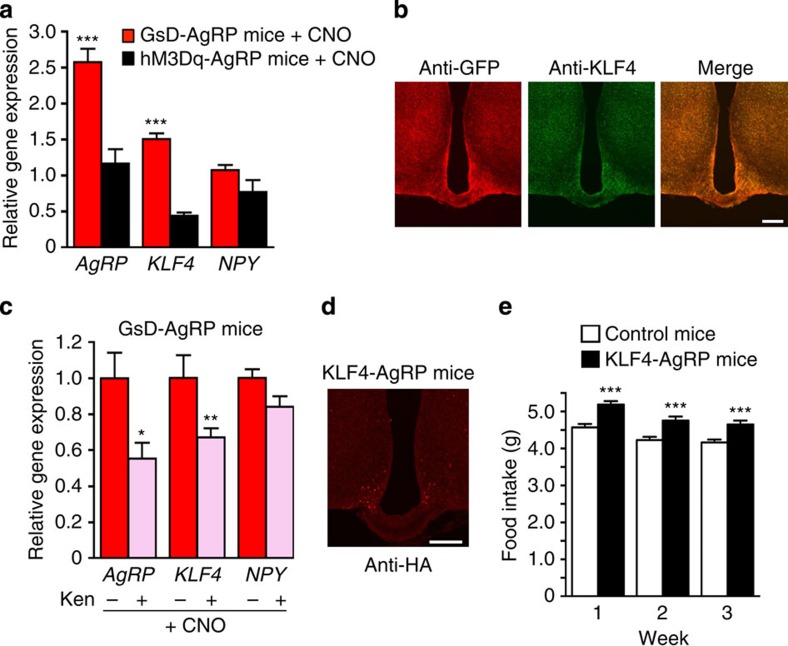
KLF4 is required for the increase in AgRP expression following GsD stimulation in AgRP neurons. (**a**) Differential effects on hypothalamic *KLF4*, *AgRP* and *NPY* mRNA expression following hM3Dq or GsD stimulation in AgRP neurons. GsD-AgRP and hM3Dq-AgRP mice were treated with CNO (1 mg kg^−1^ i.p.), and hypothalamic RNA was prepared 2 h later. Gene expression levels were determined via real-time quantitative reverse transcription–PCR (qRT–PCR). Expression data were normalized relative to results obtained with saline-injected GsD-AgRP mice (control mice). (**b**) Activation of GsD expressed in AgRP neurons of *AgRP-ires-Cre* mice selectively stimulates KLF4 protein expression in GsD-expressing neurons. The AAV-hSyn-DIO-GsD-eGFP virus was unilaterally injected into the ARC of *AgRP-ires-Cre* (right side). The GsD receptor was detected by using an anti-GFP antibody that recognizes the eGFP tag fused to the C terminus of GsD. Hypothalamic slices were processed for immunostaining studies 1 h after CNO administration (1 mg kg^−1^ i.p.). Scale bar, 200 μm. (**c**) Effects of kenpaullone (Ken), an inhibitor of *KLF4* expression, on CNO-dependent changes in gene expression in GsD-AgRP mice. GsD-AgRP mice were first injected (i.c.v.) with either saline (−) or Ken (2 μg). Thirty minutes later, all mice were injected with CNO (1 mg kg^−1^ i.p.). Hypothalamic RNA was isolated and processed for qRT–PCR studies 2 h after CNO treatment. Experiments were carried out with 11- to 17-week-old male mice. Data are given as means±s.e.m. (*n*=3–5 mice per group).*P<0.05, **P<0.01, ***P<0.001, as compared with saline-treated GsD-AgRP mice (**a**) or GsD-AgRP mice pretreated with i.c.v. saline (**c**), respectively. (**d**) Immunohistochemical detection of KLF4 expression in AgRP neurons of *AgRP-ires-Cre* mice injected with the AAV-hSyn-DIO-KLF4 virus. KLF4 was visualized by using an anti-HA antibody that recognizes the HA tag that was fused to the C terminus of KLF4. Scale bar, 200 μm. (**e**) Selective expression of KLF4 in AgRP neurons leads to enhanced food intake. To obtain KLF4-AgRP and control mice, *AgRP-ires-Cre* mice were injected bilaterally with the AAV-hSyn-DIO-KLF4 virus or the AAV-hSyn-DIO-mCherry virus, respectively. Experiments were carried out with 10-week-old male mice. Data are given as means±s.e.m. (*n*=6 mice per group).***P<0.001, as compared with the control group (**a**,**c**,**e**, Student's *t*-test).

After having demonstrated that AgRP is essential for the GsD-mediated increases in food intake, we next examined whether hypothalamic *AgRP* expression levels were altered in CNO-treated GsD-AgRP mice. Specifically, we injected GsD-AgRP mice with CNO (1 mg kg^−1^ i.p) and prepared hypothalamic RNA 2 h later. For control purposes, we carried out similar experiments with hM3Dq-AgRP mice. We found that CNO treatment of GsD-AgRP mice led to a robust (∼2.5-fold) increase in hypothalamic *AgRP* expression, as compared with saline-injected GsD-AgRP control mice (Fig. 7a). In contrast, under the same experimental conditions, hypothalamic *AgRP* expression levels remained unchanged in hM3Dq-AgRP mice (Fig. 7a). Hypothalamic *NPY* expression was not significantly affected by CNO treatment of either GsD-AgRP or hM3Dq-AgRP mice (Fig. 7a), indicating that activation of GsD in AgRP neurons selectively upregulates *AgRP* transcript levels.

Krüppel-like factor 4 (KLF4) is a zinc finger transcription factor, which is expressed in various tissues and modulates many developmental processes and biological functions^30^,^31^. Interestingly, recent studies have shown that drugs that increase intracellular cAMP levels promote *KLF4* expression in different cell types^32^,^33^, indicative of a possible link between enhanced G_s_ signalling and increased *KLF4* expression. Moreover, Ilnytska *et al*.^34^ recently demonstrated that KLF4 is a potent activator of the *AgRP* promoter. We therefore speculated that activation of the GsD designer receptor in AgRP neurons might lead to an increase in hypothalamic *KLF4* expression. In agreement with this hypothesis, we found that CNO treatment (1 mg kg^−1^ i.p) of GsD-AgRP mice resulted in a significant increase in hypothalamic *KLF4* expression levels (by ∼50%, as compared with saline-injected control mice; hypothalamic RNA was prepared 2 h after CNO injections; Fig. 7a). In contrast, CNO treatment of hM3Dq-AgRP mice did not lead to an increase but rather a reduction in hypothalamic *KLF4* expression levels (Fig. 7a).

To investigate whether the GsD-mediated stimulation of *KLF4* mRNA expression also led to increased levels of KLF4 protein, we microinjected the AAV-hSyn-DIO-GsD-eGFP virus unilaterally into the ARC of *AgRP-ires-Cre* mice (right side), leading to the expression of the GsD designer receptor in AgRP neurons of the AAV-injected side only (Fig. 7b). Treatment of the AAV-injected mice with CNO (1 mg kg^−1^ i.p.) resulted in a robust increase in KLF4 protein expression in AgRP neurons expressing the GsD receptor, measured 1 h after CNO administration (Fig. 7b, right side). Only weak KLF4 staining was detected on the side that did not express the GsD receptor (Fig. 7b, left side).

### KLF4 overexpression in AgRP neurons promotes food intake

To demonstrate that overexpression of KLF4 in AgRP neurons promotes food intake, we injected the AAV-hSyn-DIO-KLF4 virus bilaterally into the ARC of AgRP-*ires-Cre* mice, resulting in the selective expression of KLF4 in AgRP neurons (Fig. 7d). Strikingly, the AAV-hSyn-DIO-KLF4-injected mice (KLF4-AgRP mice) consumed significantly more food than the control mice injected with the AAV-hSyn-DIO-mCherry control virus (Fig. 7e).

### GsD-mediated increase in *AgRP* expression requires KLF4

We next studied whether enhanced KLF4 expression is required for the observed GsD-mediated increase in *AgRP* transcription. To test this hypothesis, we took advantage of the availability of a compound called kenpaullone (Ken), a suppressor of *KLF4* transcription^35^. Consistent with our hypothesis, we found that pretreatment of GsD-AgRP mice with Ken (2 μg i.c.v.) led to a pronounced reduction in CNO-mediated increases in both *KLF4* and *AgRP* hypothalamic expression levels (Fig. 7c). In this figure, gene expression levels are displayed normalized relative to those obtained with CNO-treated GsD-AgRP mice that had been pretreated with i.c.v. saline rather than Ken. Ken pretreatment had no significant effect on hypothalamic NPY expression levels in CNO-injected GsD-AgRP mice (Fig. 7c). These data support the concept that GsD-mediated stimulation of *AgRP* expression requires an increase in KLF4 activity.

### The *KLF4* promoter is a direct target of activated CREB

To examine whether the *KLF4* promoter is directly activated by CREB, we carried out a series of *KLF4* promoter–luciferase reporter assays (Supplementary Fig. 6a). The mouse *KLF4* promoter contains two half CREB-responsive element (CRE) sites (CGTCA) that form a cluster within 300 nucleotides upstream of the transcription start (Supplementary Fig. 6c)^36^. We found that mutational modification of the first half CRE site (−269 site) had no significant effect on forskolin-mediated stimulation of *KLF4* promoter activity. In contrast, mutation of the second half CRE site (−232 site) or of both half CRE sites virtually abolished this response (Supplementary Fig. 6b), indicating that only the second half CRE site is transcriptionally relevant. Taken together, these data strongly suggest that the *KLF4* promoter is a direct target of activated CREB.

### PAC_1_ receptors promote KLF4-dependent *AgRP* expression

We next examined whether stimulation of endogenously expressed G_s_-coupled receptors resulted in cellular actions similar to those observed with the GsD designer receptor. Initially, we carried out studies with hypothalamic GT1-7 cells^37^, which are known to release AgRP depending on their energy status^38^. We found that incubation of GT1-7 cells with forskolin (10 μM) led to a time-dependent increase in the expression of both *AgRP* and *KLF4* (Supplementary Fig. 7). These forskolin-induced effects could be completely blocked by pretreatment of cells with the PKA inhibitor, H89 (Supplementary Fig. 7), strongly suggesting that cAMP-mediated activation of PKA promotes *AgRP* and *KLF4* expression.

**Figure 8:**
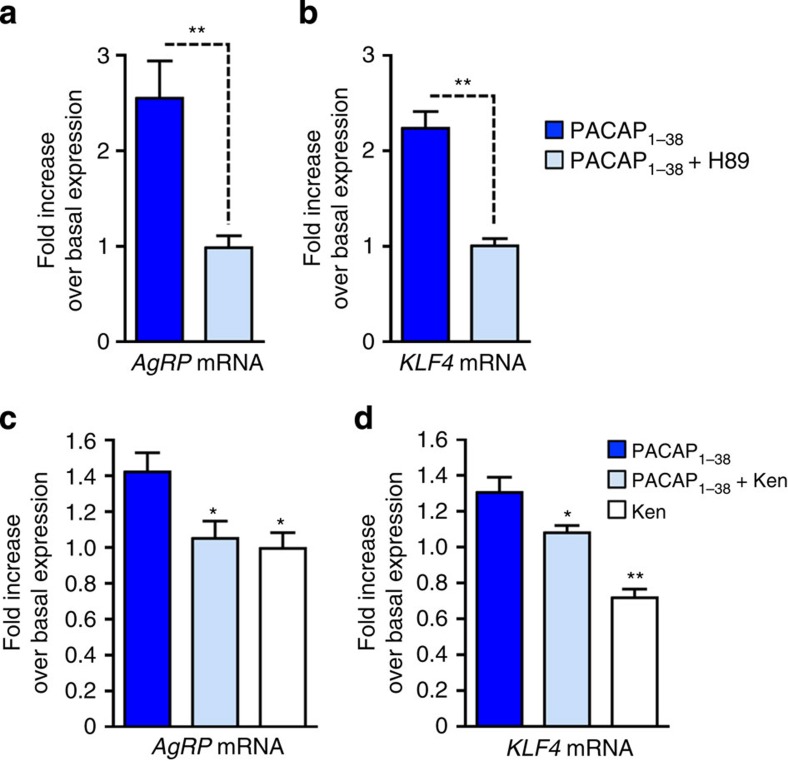
PACAP promotes *AgRP* expression in a KLF4-dependent manner. (**a**,**b**) Effect of PACAP_1–38_ (1 μM) on *AgRP* (**a**) and *KLF4* (**b**) mRNA expression in GT1-7 cells. GT1-7 cells were incubated for 2 h at 37 °C with PACAP_1–38_, either in the absence or presence of H89 (75 μM), a PKA inhibitor. (**c**,**d**) Effects of Ken, an inhibitor of *KLF4* expression, on PACAP_1–38_-induced increases in *AgRP* (**c**) and *KLF4* (**d**) expression in GT1-7 cells. GT1-7 cells were incubated for 2 h at 37 °C with either PACAP_1–38_ (1 μM), PACAP_1–38_ plus Ken (5 μM) or Ken alone (5 μM). Data are given as means±s.e.m. (*n*=3 or 4). *P<0.05, **P<0.01, ***P<0.001, as compared with the PACAP_1–38_-treated group (**a**,**b**, Student's *t*-test; **c**,**d**, one-way analysis of variance followed by Dunnett's *post hoc* test).

GT1-7 cells express various G_s_-linked GPCRs, including the PAC_1_ receptor, a pituitary adenylate cyclase-activating peptide (PACAP) receptor subtype^39^,^40^. We therefore treated GT1-7 cells with PACAP_1–38_ (1 μM) to stimulate endogenously expressed PAC_1_ receptors. PACAP_1–38_, similar to forskolin, triggered pronounced increases in the expression of both *AgRP* and *KLF4* (Fig. 8a,b). These effects were not observed in the presence of H89, indicating that the stimulatory effects of PACAP_1–38_ on *AgRP* and *KLF4* expression were due to receptor-mediated stimulation of the G_s_-cAMP-PKA cascade. Interestingly, treatment of GT1-7 cells with Ken, an inhibitor of *KLF4* transcription (Fig. 8d), completely abolished the PACAP_1–38_-induced increase in *AgRP* expression (Fig. 8c). These results support the concept that PACAP_1–38_ induces *AgRP* transcription in a *KLF4*-dependent manner.

### PAC_1_ receptors stimulate *AgRP* expression and food intake

Recent work has shown that AgRP neurons, like GT1-7 cells, express PAC_1_ receptors^21^,^41^. The potential role of these receptors in regulating food intake remains unclear. On the basis of our findings obtained with CNO-treated GsD-AgRP mice, we tested the hypothesis that selective activation of PAC_1_ receptors endogenously expressed by AgRP neurons might cause a sustained increase in food intake.

**Figure 9:**
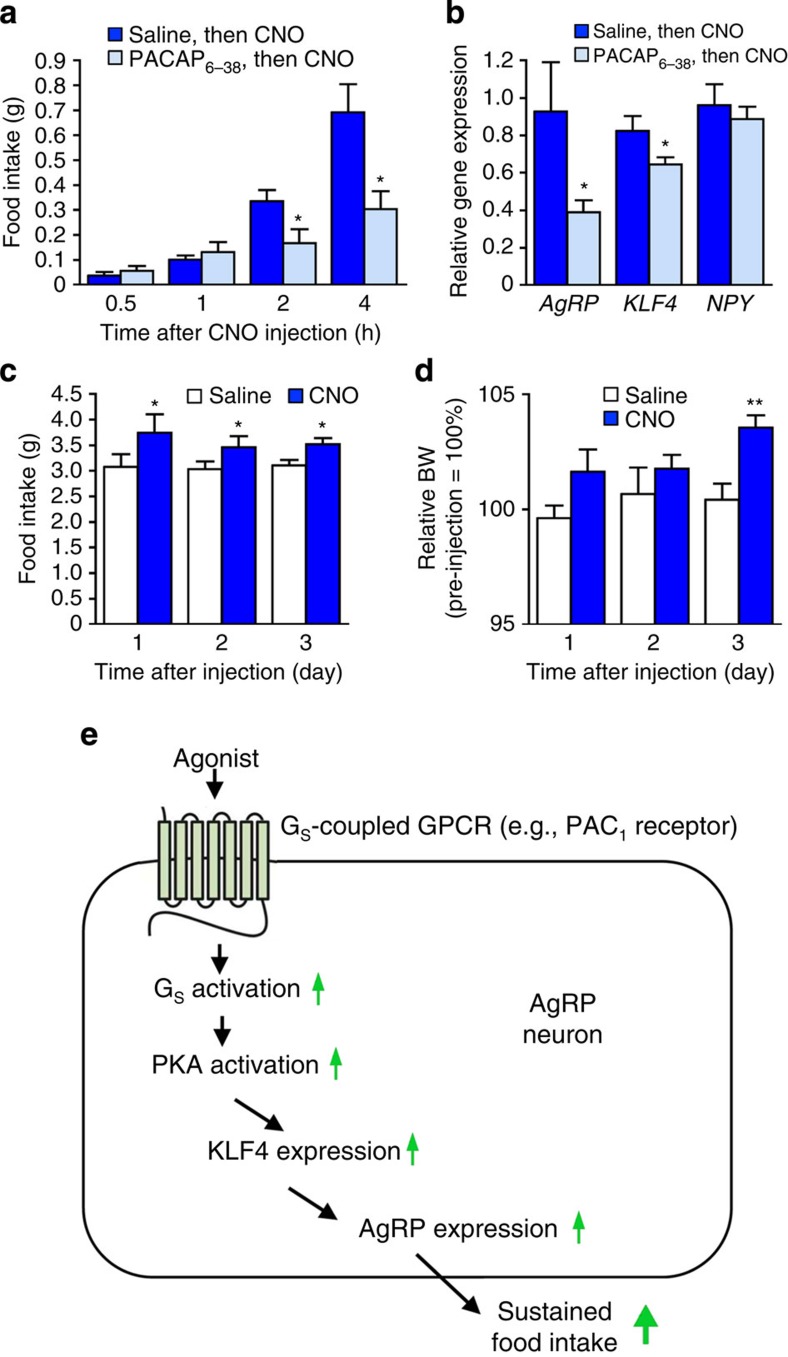
Selective activation of PVN-PACAP neurons mimics the phenotypes displayed by CNO-treated GsD-AgRP mice. All studies were carried out with mice selectively expressing hM3Dq in PACAP neurons of the PVN of *PACAP-ires-Cre* mice (hM3Dq-PACAP mice; see ref. 21). (**a**) PACAP_6–38_, a PACAP receptor antagonist, inhibits CNO-induced acute food intake in hM3Dq-PACAP mice. Mice initially received an i.c.v. injection of either saline or PACAP_6–38_ (1.2 μg). Thirty minutes later, all mice were injected with CNO (1 mg kg^−1^ i.p.). (**b**) Activation of PVN-PACAP neurons promotes hypothalamic *AgRP* and *KLF4* expression. hM3Dq-PACAP mice were injected (i.c.v.) with either saline or PACAP_6–38_ (1.2 μg). Thirty minutes later, all mice were treated with CNO (1 mg kg^−1^ i.p.). Hypothalamic RNA was isolated and processed for quantitative reverse transcription–PCR studies 2 h after CNO treatment. Expression data were normalized relative to the results obtained with CNO-treated GsD-AgRP mice. (**c**,**d**) Activation of PVN-PACAP neurons leads to long-lasting increases in food intake and increased body weight (BW). hM3Dq-PACAP mice received a single i.p. injection of either saline or CNO (1 mg kg^−1^). Changes in BW were expressed relative to pre-injection values (=100%). (**e**) Scheme summarizing the cellular pathway through which agonist-activated G_s_-coupled GPCRs expressed by AgRP neurons are predicted to promote appetite. All experiments were carried out with 10- to 16-week-old male mice. Data are given as means±s.e.m. (*n*=3–6 mice per group). *P<0.05, **P<0.01, as compared with the corresponding control group (Student's *t*-test).

To stimulate these receptors *in vivo*, we took advantage of the recent finding that PACAP-containing glutamatergic neurons in the paraventricular nucleus of the hypothalamus (PVN) are monosynaptically connected to AgRP neurons^21^. To selectively activate this neuronal pathway, we first injected the AAV-hM3Dq virus (AAV-hSyn-DIO-hM3Dq-mCherry) into the PVN of *PACAP-ires-Cre* mice (bilateral injections; use of ‘Flex Switch' technology). This strategy resulted in the selective expression of the hM3Dq designer receptor in PACAP neurons of the PVN^21^. For the sake of brevity, we refer to these mice as hM3Dq-PACAP mice. To activate the hM3Dq-expressing PACAP neurons *in vivo*, we injected hM3Dq-PACAP mice with a single dose of CNO (1 mg kg^−1^ i.p.). Before CNO injections, hM3Dq-PACAP mice received an i.c.v. injection of either saline or PACAP_6–38_ (1.2 μg), a potent PAC_1_ receptor antagonist. CNO treatment of hM3Dq-PACAP mice pre-injected with saline resulted in a robust, time-dependent increase in acute food take (Fig. 9a). Strikingly, this response was greatly reduced in CNO-injected hM3Dq-PACAP mice that had been pretreated with PACAP_6–38_ (Fig. 9a). PACAP_6–38_ had no significant effect on food intake in WT mice (Supplementary Fig. 8).

We next examined whether CNO treatment of hM3Dq-PACAP mice resulted in changes in the hypothalamic transcript levels of *AgRP*, *KLF4* and *NPY*. Specifically, hM3Dq-PACAP mice that had been pretreated i.c.v. with either saline or PACAP_6–38_ (1.2 μg) were injected with CNO (1 mg kg^−1^ i.p.), and hypothalami were isolated and processed for quantitative reverse transcription–PCR studies 2 h later. In the absence of PACAP_6–38_, CNO treatment led to significant increases in hypothalamic *AgRP* and *KLF4* expression levels, which were similar in magnitude to those observed with CNO-treated GsD-AgRP mice (Fig. 9b). In contrast, in hM3Dq-PACAP mice that had been pretreated with PACAP_6–38_, CNO-induced *AgRP* and *KLF4* expression (but not *NPY* expression) was significantly suppressed (Fig. 9b). These results suggest that activation of PAC_1_ receptors endogenously expressed by AgRP neurons promotes food intake by stimulating the KLF4-AgRP axis.

Finally, we investigated whether CNO treatment of hM3Dq-PACAP mice had long-lasting effects on food intake, as observed with CNO-injected GsD-AgRP mice. We found that a single injection of CNO (1 mg kg^−1^ i.p.) stimulated food intake for 3 days, similar to the results obtained with GsD-AgRP mice (Figs 3b and 9c). The long-lasting stimulatory effect of CNO on food intake was accompanied by an increase in body weight (note that this effect was highly significant on day 3 after CNO injection; Fig. 9d). Taken together, these data strongly suggest that activation of PAC_1_ receptors endogenously expressed by AgRP neurons mimics the sustained orexigenic effect triggered by GsD signalling in these neurons.

### Fasting promotes pCREB expression in AgRP neurons

**Figure 10:**
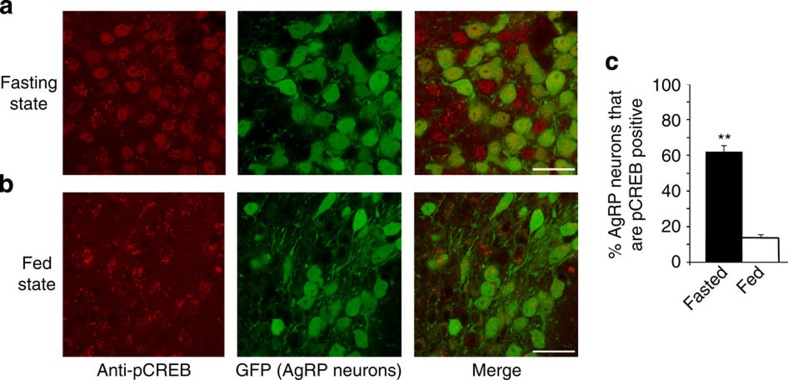
Fasting-induced increase in pCREB expression in AgRP neurons of the ARC. (**a**) Representative confocal images of hypothalamic sections obtained from *Npy-GFP* mice after a 20-h fast. pCREB-expressing cells are visualized by an anti-pCREB antibody (red stain, left panel). AgRP (NPY)-positive neurons are visualized by green GFP fluorescence (centre panel). The right panel represents a merged image indicating that most AgRP neurons are pCREB positive. (**b**) Images corresponding to the ones shown in **a** were obtained with freely fed *Npy-GFP* mice, indicating a striking reduction in pCREB-positive AgRP neurons. (**c**) Quantification of confocal images. The percentage of AgRP-positive neurons (as assessed by GFP fluorescence) staining positive for pCREB was calculated. Data are expressed as means±s.e.m. (13–17 sections from three different adult male mice per group were analysed). **P<0.01 (Student's *t*-test). Scale bar, 20 μm.

To explore whether G_s_-pCREB signalling in AgRP neurons is sensitive to altered metabolic conditions such as fasting when AgRP neurons show increased activity, we studied pCREB expression in AgRP neurons of *Npy-hrGFP* mice (*Npy-GFP* mice)^42^. This mouse line expresses GFP selectively in NPY/AgRP neurons^42^. Specifically, we prepared hypothalamic sections from *Npy-GFP* mice that had either free access to food or had been fasted for 20 h. AgRP neurons were identified by GFP fluorescence (green) while pCREB expression was probed with an anti-pCREB antibody (red fluorescence; Fig. 10 and Supplementary Fig. 9).We found that fasted mice showed a marked increase in the number of AgRP neurons staining positive for pCREB (Fig. 10c). This observation suggests that pCREB expression in AgRP neurons is likely to be of physiological relevance.

## Discussion

Food intake studies showed that treatment of GsD-AgRP mice with a single dose of CNO resulted in a long-lasting (for several days) orexigenic effect that was associated with a significant increase in body weight (Fig. 3a–c). In contrast, the stimulatory effect observed with CNO-treated hM3Dq-AgRP mice lasted only for a single day (Fig. 3d–f), most likely reflecting differences in cellular activity caused by the activation of G_s_- versus G_q_-dependent signalling pathways (Figs 1c and 2).

AgRP neurons store and release the neuropeptides AgRP and NPY, as well as the biogenic amine, GABA, all of which promote food intake^4^. Using a combined genetic/pharmacological approach, we demonstrated that NPY and GABA release do not contribute to a significant extent to the orexigenic effects mediated by the GsD designer receptor expressed by AgRP neurons (Fig. 5). In contrast, the GsD-mediated increase in food intake was completely abolished by i.c.v. treatment of GsD-AgRP mice with an anti-AgRP antibody (Fig. 4c). These data clearly indicate that the ability of the G_s_-coupled designer receptor to promote food intake is mediated by the release of AgRP.

In striking contrast, the anti-AgRP antibody had no significant effect on the CNO-induced increase in food intake observed with hM3Dq-AgRP mice (Fig. 4d). In agreement with this observation, Krashes *et al*.^5^ recently reported that the release of either NPY or GABA alone is sufficient to drive acute feeding in CNO-treated hM3Dq-AgRP mice and that AgRP does not contribute to this response.

Given the key role of AgRP in mediating the orexigenic effects displayed by CNO-treated GsD-AgRP mice, we speculated that CNO stimulation of GsD might affect AgRP expression. Consistent with this hypothesis, CNO treatment of GsD-AgRP mice resulted in a robust increase in hypothalamic *AgRP* transcript levels (Fig. 7a). In contrast, hypothalamic *AgRP* expression remained unaffected in CNO-injected hM3Dq-AgRP mice (Fig. 7a). It is likely that the increase in GsD-mediated *AgRP* expression represents a key factor contributing to the long-lasting orexigenic effects observed with CNO-treated GsD-AgRP mice.

Since drugs that enhance the production of cAMP can stimulate *KLF4* expression in different cell types^32^,^33^, we tested the hypothesis that GsD-mediated stimulation of *KLF4* expression is required for the GsD-dependent increase in *AgRP* levels observed with GsD-AgRP mice. We first showed that CNO treatment of GsD-AgRP mice led to a significant increase in hypothalamic *KLF4* mRNA levels and a pronounced increase in KLF4 protein levels in AgRP neurons expressing the GsD designer receptor (Fig. 7a,b). Studies with a chemical inhibitor of *KLF4* transcription^35^ indicated that the GsD-mediated increase in hypothalamic *AgRP* expression requires KLF4 (Fig. 7c). Importantly, overexpression of KLF4 in AgRP neurons led to a significant increase in food intake (Fig. 7d,e). Taken together, these data strongly support the concept that activation of the G_s_-linked designer receptor (GsD) promotes food intake by stimulating *KLF4*-dependent expression of *AgRP*.

Studies with cultured hypothalamic cells demonstrated that ligand-induced activation of an endogenously expressed G_s_-linked receptor, the PAC_1_ receptor subtype, also promoted *KLF4* and *AgRP* expression, similar to the findings obtained with CNO-treated GsD-AgRP mice. Additional studies indicated that these responses were PKA dependent and that increases in *KLF4* expression triggered the upregulation of *AgRP* expression (Fig. 8). Collectively, these data strongly support the concept that activation of G_s_-coupled receptors endogenously expressed by AgRP neurons (for example, PAC_1_ receptors) stimulates a PKA-dependent signalling cascade that promotes the expression of *KLF4* and *AgRP* in a sequential fashion (Fig. 9).

A recent study^43^ reported that overexpression of KLF4 in the ARC of rats abolished the anorectic actions of leptin. Taken together, these findings indicate that KLF4 represents an important central regulator of the activity of hypothalamic AgRP neurons.

In agreement with the *in vitro* studies, *in vivo* experiments demonstrated that the activation of G_s_-linked PAC_1_ receptors endogenously expressed by AgRP neurons closely mimicked the stimulatory effects on food intake and body weight observed with CNO-treated GsD-AgRP mice (Figs 3 and 9). In this set of experiments, we used a chemogenetic strategy to selectively activate the PACAP-containing neurons of the PVN that are monosynaptically connected to AgRP neurons^21^. Importantly, the increase in food intake observed after activation of this pathway could be greatly reduced by pretreatment of mice with PACAP_6–38_, a PAC_1_ receptor antagonist (Fig. 9a). These data strongly support the concept that stimulation of PAC_1_ receptors or other G_s_-coupled receptors endogenously expressed by AgRP neurons leads to a sustained increase in food intake (Fig. 9e). Interestingly, microarray data have shown that AgRP neurons express additional G_s_-coupled GPCRs, besides the PAC_1_ receptor, including calcitonin and MC_3_ melanocortin receptors (Geo dataset GSE45858; also see ref. 44). It is likely that signalling through all of these receptors contributes to G_s_-mediated AgRP release.

As pointed out at the beginning of the Discussion section, CNO treatment did not depolarize GsD-AgRP neurons in *ex vivo* whole-cell patch clamp experiments (Fig. 1c,d). However, this finding does not exclude the possibility that CNO-mediated stimulation of GsD affects the activity of downstream signalling molecules and/or ion channels that facilitate the activation of AgRP neurons *in vivo* where these neurons are exposed to a multitude of hormones and neurotransmitters. Consistent with this concept, activation of the G_s_-cAMP signalling pathway by G_s_-coupled receptors has been shown to promote neuronal excitability^45^,^46^,^47^. It is likely that the sustained orexigenic effect observed with CNO-treated GsD-AgRP mice is due to cAMP-PKA-mediated changes at the transcriptional level (Figs 7, 8, 9) and/or G_s_-dependent changes in synaptic plasticity^48^, which remain to be explored.

Our findings raise the possibility that drugs that block the activity of G_s_-coupled receptors endogenously expressed by AgRP neurons or that interfere with G_s_-dependent downstream signalling pathways might become useful as appetite-suppressing drugs. The identification of the complete set of GPCRs expressed by AgRP neurons under physiological and pathophysiological conditions should provide a rational basis for this approach.

## Methods

### Mice

The generation of *AgRP-ires-Cre* mice (ref. 49), *PACAP-ires-Cre* (ref. 21), *Vgat*_*flox/flox*_ (ref. 49), *NPY-hrGFP* (ref. 42) and *Agrp-ires-Cre; R26-loxSTOPlox-L10-GFP* (L10-GFP mice^21^) has been described previously. In L10-GFP mice, eGFP fused to the L10-ribosomal subunit is selectively expressed, in a Cre-dependent fashion, in AgRP neurons^21^. *A*^*y*^ mice (strain name: B6.Cg-A^y^/J; stock number: 000021) were purchased from the Jackson Laboratory (Bar Harbor, ME). All experiments were conducted according to the US National Institutes of Health Guidelines for Animal Research and were approved by the NIDDK Institutional Animal Care and Use Committee.

### Mouse maintenance and diet

Mice were fed *ad libitum* and kept on a 12-h light, 12-h dark cycle. Unless stated otherwise, all experiments were carried out with male littermates that were 10–16 weeks old and maintained on a standard NIH mouse chow (∼4% (w/w) fat content).

### Generation of AAV-hSyn-DIO vectors

The generation of the AAV-hSyn-DIO-hM3Dq-mCherry and AAV-hSyn-DIO-mCherry viral vectors has been described previously^19^. To generate the AAV-hSyn-DIO-GsD-eGFP vector, we replaced the hM3Dq-mCherry coding sequence in the pAAV-hSyn-DIO hM3Dq-mCherry plasmid (a kind gift by Dr Bryan Roth, UNC, Chapel Hill, NC) with the HA-GsD-eGFP coding sequence^14^. The AAV-hSyn-DIO-rM3D(Gs)-mCherry virus was provided by the UNC Vector Core (Chapel Hill, NC). This latter virus is identical with the AAV-hSyn-DIO-GsD-eGFP virus except for the different fluorescence tags (mCherry versus eGFP, respectively). To generate the AAV-hSyn-DIO-KLF4 vector, we first fused the human influenza hemagglutinin (HA) tag sequence to the KLF4 coding sequence (Addgene plasmid #15920) by using a PCR strategy. Then, the KLF4-HA coding sequence was cloned into the pAAV-hSyn-DIO vector. All AAVs were packaged in serotype 8 by the UNC Vector Core.

### Stereotaxic injections

Male mice that were at least 6 weeks old were anaesthetized with isoflurane, and placed into a stereotaxic apparatus (David Kopf Instruments, model 940A with 923B mouse gas aesthesia head holder). The skull was exposed via a small incision and a small hole was drilled (0.45-mm drill bit) into the skull for the injection of AAVs. A Hamilton 10-μl syringe with a 30- or 33-gauge blunt-end needle was inserted into the brain for virus delivery. Postoperative analgesia was provided (ketoprofen, 5 mg kg^−1^ subcutaneous). Bilateral injections (200 nl of AAV) were made in the ARC. The coordinates were (from bregma) as follows: anterior–posterior, −1.46 mm; lateral (from midline), ±0.3 mm; dorsal–ventral, −5.80 mm). In a similar fashion, bilateral injections were made in the PVN. The coordinates were (from bregma) as follows: anterior–posterior, −0.82 mm; lateral (from midline), ±0.25 mm; dorsal–ventral, −4.80 mm (from the surface of the skull). Mice were allowed to recover for 10 days before the start of feeding experiments.

### Immunohistochemistry and imaging studies

Mice were perfused with 4% paraformaldehyde in 0.1 M phosphate buffer fixative (pH 7.4). Tissue was post-fixed in this solution overnight and transferred to 20% sucrose in 1 × PBS. Hypothalamic slices (20–30-μm thick) were incubated overnight at 4 °C with primary antibodies diluted in PBS, supplemented with 1% BSA and 0.1% Triton X-100. Slices were then washed three times and incubated with fluorophore-conjugated secondary antibodies for 2 h at room temperature. Slices were rinsed three times in PBS containing 0.1% Triton X-100 and then mounted for imaging using Antifade Reagent (SlowFade Gold with DAPI, S36939; Life Technologies, Grand Island, NY) or Vectashield technology (Vector Labs, Burlingame, CA). Fluorescence images were taken with a Zeiss Imager D1 fluorescent microscope and a Zeiss LSM-700 confocal microscope.

### Quantification of c-fos and pCREB expression

To quantitate CNO-induced changes in c-fos and pCREB expression in DREADD-expressing AgRP neurons or changes in pCREB expression in AgRP neurons of fed and fasted *Npy-GFP* mice^42^, we carried out a series of immunohistochemical studies. CNO (1 mg kg^−1^)-injected GsD-AgRP and hM3Dq-AgRP mice were killed 2 h after drug treatment. For fasting experiments, *Npy-GFP* mice were killed at 9:00 in either the *ad lib* fed state or after a 20-h fast (food was removed at 12:00 on the previous day). Hypothalamic sections were prepared and processed for immunohistochemical staining as described above. AgRP neurons/DREADD-expressing AgRP neurons staining positive for pCREB or c-fos were visualized and counted using confocal images taken under × 20 and × 40 power of magnification. Fluorescent images were captured using a Zeiss LSM-700 confocal microscope. Cells were counted by an observer that was blinded to mouse treatment or genotype of the mice. Data are expressed as the percentage of all AgRP neurons staining positive for pCREB (experiments with *Npy-GFP* mice) or as the percentage of DREADD-expressing AgRP neurons staining positive for pCREB or c-fos, respectively. The antibodies used are listed in Supplementary Table 2.

### I.c.v. injections

For i.c.v. injections of drugs or antibodies, mice were anaesthetized with 2–3% isoflurane and placed into a stereotaxic apparatus (see ‘Stereotaxic injections'). The surface of the skull was exposed with sterile surgical instruments, and a small hole was drilled through the skull and a guide cannula was placed into the lateral ventricle via the stereotaxic manipulator. For i.c.v. injections, the following coordinates were used (from bregma): anterior–posterior, −0.34 mm; lateral (from midline), +1 mm; dorsal–ventral, −2.3 mm (from the surface of the skull). The guide cannula (Plastic One; C315GS-2/SP, 2.6 mm with 2 mm pedestal; Roanoke, VA) was placed and secured using Loctite 454 Glue. After a 2-week recovery period, a small injection cannula (Plastic One; C315IS-2/SPC, fit 2.6 mm C315GS-2 with 1 mm projection) attached to a catheter and a Hamilton syringe was inserted into the guide cannula. The injection cannula protruded 1 mm beyond the end of the guide cannula into the ventricle. Drugs or antibodies were delivered slowly in a 2-μl volume via the injection cannula. After an additional 7 min, the injection cannula was removed and capped with a dummy cannula (C315FDS-2; Plastic One). The following drugs or antibodies were delivered via this route: AgRP_83–132_ (1 μg; Phoenix Pharmaceuticals), anti-AgRP purified IgG antibody (3 μg; Phoenix Pharmaceuticals), BIBO3304 (3.3 μg; Tocris, MO), CNO (0.1, 0.3, 0.5 or 1 μg), kenpaullone (2 μg; Sigma-Aldrich), NPY (0.85 μg; Phoenix Pharmaceuticals) and PACAP_6–38_ (1.2 μg; Phoenix Pharmaceuticals).

### Assessment of neuropeptide and *KLF4* mRNA levels

*AgRP*, *KLF4* and *NPY* mRNA levels were measured using RNA prepared from cultured GT1-7 cells or from mouse hypothalamic tissue. One day before the assay, GT1-7 cells were seeded into six-well plates at a density of 1 × 10^6^ cell per dish and cultured in DMEM containing 10% fetal bovine serum and penicillin (100 units per ml)/streptomycin (100 μg ml^−1^) in a humidified atmosphere of 5% CO_2_/95% O_2_ at 37 °C. On the day of the assay, the medium was replaced with fresh DMEM. One hour later, GT1-7 cells were incubated for 2 h at 37 °C with the following compounds (final concentrations): PACAP_1–38_ (1 μM; Phoenix Pharmaceuticals), H89 (75 μM; Sigma-Aldrich) or kenpaullone (5 μM; K3888, Sigma-Aldrich). Total RNA was then isolated using the RNeasy Mini Kit (Qiagen) and treated with DNase I for 15 min at 30 °C. Reverse transcription was performed using the SuperScript III first strand synthesis kit (Invitrogen) and gene expression levels were measured by monitoring SYBR green fluorescence intensity over time using a Bio-Rad CFX96 Real Time System C1000 Touch Thermal Cycler (Bio-Rad). Each PCR reaction (final volume: 20 μl) consisted of cDNA (1 μg of initial RNA sample), 10 μl of SYBR Green PCR Master Mix (Applied Biosystems) and 100 nM of each PCR primer. For each primer pair, quantitative reverse transcription–PCR reactions were performed in duplicate using a 96-well plate format. Primers for *β*-*actin*, *AgRP*, *KLF4* and *NPY* were selected from previously validated primer sets (see table below). PCR cycling conditions were as follows: 50 °C for 2 min, 95 °C for 10 min; and 40 cycles at 95 °C for 15 s and 60 °C for 1 min, respectively. The expression of *β-actin* RNA served as an internal control. The results were expressed as fold change in expression of a particular RNA transcript relative to *β-actin* RNA expression between untreated and drug-treated cells.

In some experiments, AAV-injected mice (hM3Dq-AgRP, GsD-AgRP or hM3Dq-PACAP mice) were injected i.p. with saline or CNO (1 mg kg^−1^). In a subset of experiments, i.c.v. injections of kenpaullone or PACAP_6–38_ were performed 30–45 min before i.p. saline or CNO injections. Two hours after the administration of CNO (or saline), RNA was extracted from mouse hypothalami using the QIAzol Lysis reagent (Qiagen). cDNA synthesis and qPCR studies were performed as described in the previous paragraph. Experimental data were expressed as fold change in expression of a particular RNA transcript relative to *β-actin* RNA expression between a specific test group and the corresponding control group (for primer sequences, see Supplementary Table 3).

### Electrophysiology

To obtain brain slices that contain the ARC, mice (age: ∼10 weeks) were decapitated under isoflurane anaesthesia, and brains were rapidly removed and placed in ice-cold sucrose aCSF (composition in mM): 194 sucrose, 20 NaCl, 4.4 KCl, 2 CaCl_2_, 1 MgCl_2_, 1.2 NaH_2_PO_4_, 10.0 glucose and 26.0 NaHCO_3_, saturated with 95% O_2_/5% CO_2_ gas. The 300-μm sections were sliced using a Leica VT1000 vibratome (Wetzlar, Germany).

For whole-cell patch clamp electrophysiological recordings, brain slices containing the ARC were incubated at ∼30 °C in a heated, oxygenated holding chamber containing aCSF (composition in mM): 124 NaCl, 4.4 KCl, 2 CaCl_2_, 1.2 MgSO_4_, 1 NaH_2_PO_4_, 10.0 glucose and 26.0 NaHCO_3_, and then transferred to a submerged recording chamber maintained at ∼30 °C (Warner Instruments, Hamden, CT). Recording electrodes (3–5 MΩ) were pulled with a Flaming-Brown Micropipette Puller (Sutter Instruments, Novato, CA) using thin-walled borosilicate glass capillaries. These recording electrodes were filled with a solution of the following composition (in mM): 135 K^+^-gluconate, 5 NaCl, 2 MgCl_2_, 10 HEPES, 0.6 EGTA, 4 Na_2_APT and 0.4 Na_2_GPT. To block neural firing, tetrodotoxin (0.5 μM) was added to the perfusing aCSF solutions described above. Signals were acquired via a Multiclamp 700B amplifier (Molecular Devices, Sunnyvale, CA), digitized at 20 kHz, filtered at 3 kHz and analysed using Clampfit 10.4 software (Molecular Devices). Input resistance and access resistance were monitored before and after each experiment. Experiments in which changes in access resistance were >20% were not included in the data analysis. A maximum of two neurons were analysed per mouse. Membrane potential shift was compared between a 4-min bin average during baseline and a 4-min bin average at the end of drug application. *NPY-hrGFP* mice were used to visualize AgRP neurons. In the case of GsD-AgRP and hM3Dq-AgRP mice, AgRP neurons were identified by mCherry fluorescence (both GsD and hM3Dq were fused to mCherry at their C termini).

### Food intake measurements

Mice (10- to 16-week-old males) were housed singly for at least 1 week before food intake measurements. CNO or other drugs were injected, either i.p. or i.c.v., between 9:00 and 10:00. After drug treatment, mice were placed in new cages with 5–6 food pellets (2.5–4 g per pellet) of standard mouse chow. Food intake was measured 0.5, 1, 2, 4, 24, 48 and 72 h after drug injections.

### Measurement of AgRP release from hypothalamic slices

The release of AgRP from mouse hypothalamic slices was measured *ex vivo*, in a fashion similar to that described by Enriori *et al*.^50^ The 2-mm-thick hypothalamic slices containing the ARC and PVH were prepared from GsD-AgRP and mCherry-AgRP control mice (*AgRP-ires-Cre* mice that had been injected bilaterally with the AAV-hSyn-DIO-mCherry virus). The slices were then incubated in aCSF plus 1.7 μl ml^−1^ of protease inhibitor cocktail (Sigma) and equilibrated with 95% O_2_ and 5% CO_2_ at 37 °C for 1 h. Hypothalamic slices were then incubated for 1 h in aCSF to determine basal AgRP release. Subsequently, slices were incubated for another 1 h period in the presence of CNO (15 μM). Tissue viability was verified by exposure to 56 mM KCl for 1 h. Supernatants were collected, and AgRP concentrations were determined using an AgRP fluorescence EIA kit (Phoenix Pharmaceuticals) according to the manufacturer's protocol. The amount of AgRP secreted into the medium in the presence of CNO was normalized to basal AgRP release determined during the initial aCSF incubation period.

### Generation of *KLF4* promoter–luciferase reporter constructs

The pGL3–pKLF4 luciferase reporter construct^51^ containing ∼1.5 kb of the mouse *KLF4* promoter was kindly provided by Dr John Christman (The Ohio State University Wexner Medical Center, Columbus, OH). The two half CRE sites (CGTCA) present in the *KLF4* promoter (−269 to −265 and −232 to −228, respectively) were mutated to CATGG (ref. 52) using the QuikChange Site-Directed Mutagenesis Kit (Agilent). The correctness of all constructs was verified by sequencing. The numbering of *KLF4* promoter sequences is based on the NM_010637 NCBI reference sequence.

### Luciferase reporter assays

HEK293T cells were transfected with the pGL3–pKLF4 reporter construct using Lipofectamine 2000 (Invitrogen) according to the manufacturer's instructions. Where indicated, cells were transfected with the pCMV–FLAG–ACREB construct (kindly provided by Dr Rebecca Berdeaux) or pcDNA3.1 (empty vector) to adjust for the amount of transfected DNA. All cells were co-transfected with the pACLA-RSV-β-Gal plasmid^52^, which served as internal control to normalize the luminescence signal for transfection efficiency. About 24 h after transfection, cells were incubated in 24-well plates for 5 h at 37 °C with forskolin (10 μM) or vehicle (DMSO), and luciferase assays were performed as described previously^53^.

### Statistical analysis

Data are expressed as means±s.e.m. for the indicated number of observations. The statistical tests used are indicated in the figure legends.

## Additional information

**How to cite this article:** Nakajima, K. *et al*. Gs-coupled GPCR signalling in AgRP neurons triggers sustained increase in food intake. *Nat. Commun.* 7:10268 doi: 10.1038/ncomms10268 (2016).

## Supplementary Material

Supplementary Information Supplementary Figures 1-9 and Supplementary Tables 1-3
