# Corrigendum: Gs-coupled GPCR signalling in AgRP neurons triggers sustained increase in food intake

**DOI:** 10.1038/ncomms11019

**Published:** 2016-03-03

**Authors:** Ken-ichiro Nakajima, Zhenzhong Cui, Chia Li, Jaroslawna Meister, Yinghong Cui, Ou Fu, Adam S. Smith, Shalini Jain, Bradford B. Lowell, Michael J. Krashes, Jürgen Wess

Nature Communications 7: Article number: 10268 10.1038/ncomms10268 (2016); Published: 01082016; Updated: 03032016

The financial support for this article was not fully acknowledged. The Acknowledgements should have included the following:

This work was supported by the intramural research program of the NIMH (ZIA-MH-002498-24).

