# Supplementary material for: Gs-coupled GPCR signalling in AgRP neurons triggers sustained increase in food intake
Source: Nat Commun. 2016 Jan 8;7:10268. doi: 10.1038/ncomms10268 (PMC4729878; doi:10.1038/ncomms10268)
Supplement: Supplementary Information — Supplementary Figures 1-9 and Supplementary Tables 1-3 [file ncomms10268-s1.pdf]

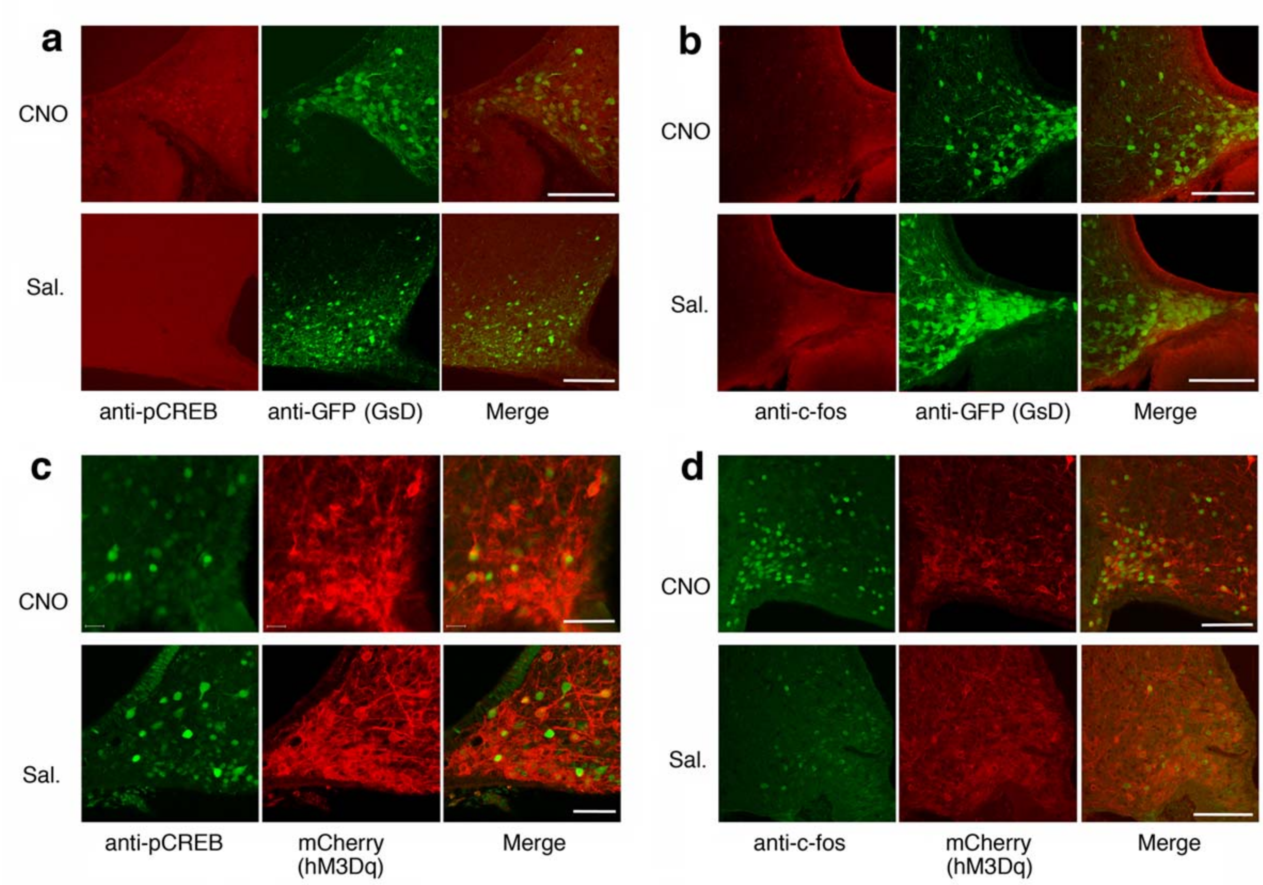


**Supplementary Figure 1.** CNO activation of the GsD designer receptor selectively promotes pCREB expression in AgRP neurons of the ARC. This figure shows representative low-magnification confocal images. (**a, b**) AAV-mediated functional expression of GsD (GsD-eGFP) in AgRP neurons. pCREB (a) or c-fos (b) expression in AgRP neurons of GsD-AgRP mice was studied after CNO (1 mg $kg^{-1}$) or saline (control) treatment. pCREB and c-fos expression were revealed by anti-pCREB- and anti-c-fos antibodies, respectively. The GsD DREADD was visualized by an anti-GFP antibody targeted at the eGFP tag fused to the C-terminus of GsD. The images show selective, CNO-mediated induction of pCREB expression in GsD-expressing AgRP neurons. (**c, d**) AAV-mediated functional expression of hM3Dq (hM3Dq-mCherry) in AgRP neurons. pCREB (c) or c-fos (d) expression in AgRP neurons of hM3Dq-AgRP mice was studied after CNO (1 mg $kg^{-1}$) or saline (control) treatment. pCREB and c-fos expression were revealed by anti-pCREB- and anti-c-fos antibodies, respectively. The hM3Dq DREADD was visualized by mCherry fluorescence (note that this DREADD contained a C-terminal mCherry tag). The images show selective, CNO-mediated induction of c-fos expression in hM3Dq-expressing AgRP neurons. Scale bars: (a, b, d), 100 μm; (c), 50 μm.

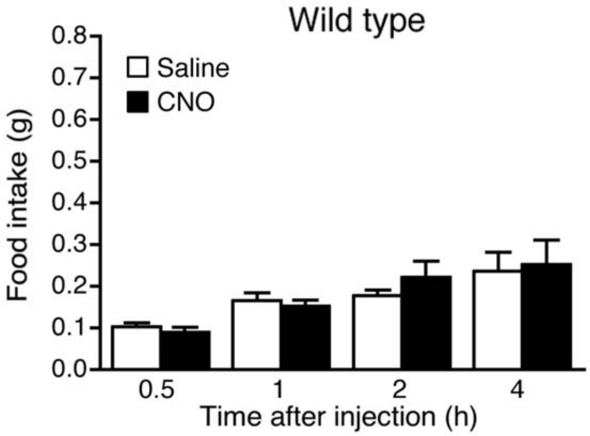


**Supplementary Figure 2.** CNO has no significant effect on food intake in WT mice. Food intake was measured after a single i.c.v. injection of either saline or CNO (1 μg) for the indicated period of time. Injections were performed between 9 to 10 a.m. Experiments were carried out with WT mice (17-week old males). Data are given as means ± s.e.m. (n=4 or 5 mice per group).

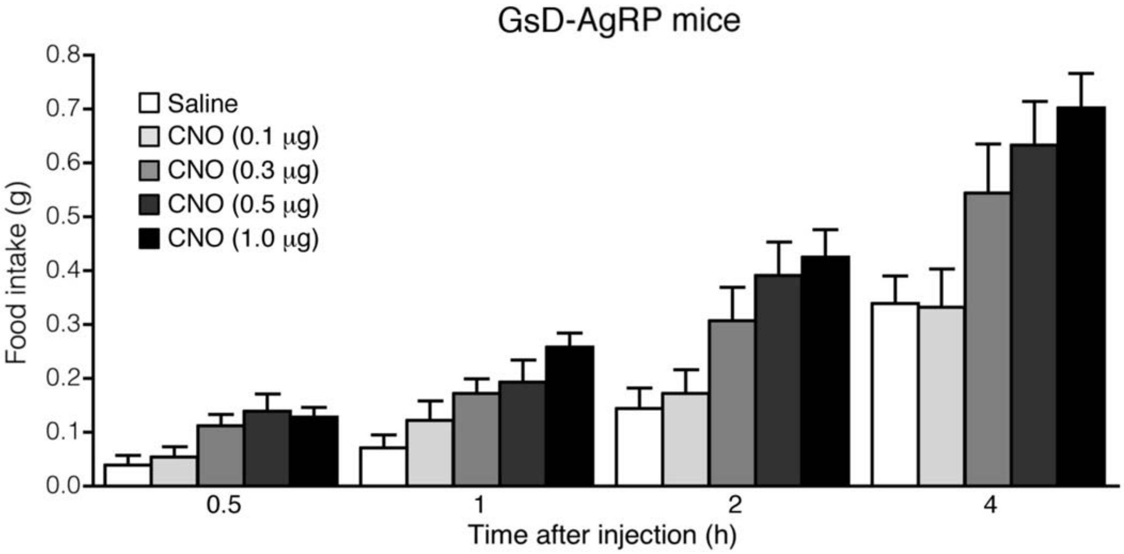


**Supplementary Figure 3.** CNO promotes food intake in GsD-AgRP mice in a dose-dependent manner. Food intake was measured after a single i.c.v. injection of either saline or CNO ( 0.1, 0.3, 0.5, or 1 μg) for the indicated time period. Injections were performed between 9 to 10 a.m. Experiments were carried out with male GsD-AgRP mice (10-16 weeks old). Data are given as means ± s.e.m. (n=4-7 mice per group).

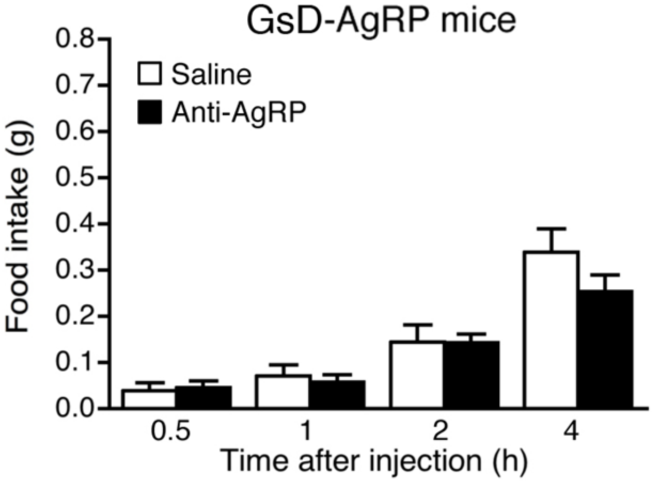


**Supplementary Figure 4.** In the absence of CNO, an anti-AgRP antibody has no significant effect on food intake in GsD-AgRP mice. Food intake was monitored after a single i.c.v. injection of either saline or an anti-AgRP antibody (1 µg) for the indicated period of time (injection time: 9 to 10 a.m.). Experiments were carried out with male GsD-AgRP mice (24 weeks old) in the absence of CNO. Data are given as means ± s.e.m. (n=6 or 7 mice per group).

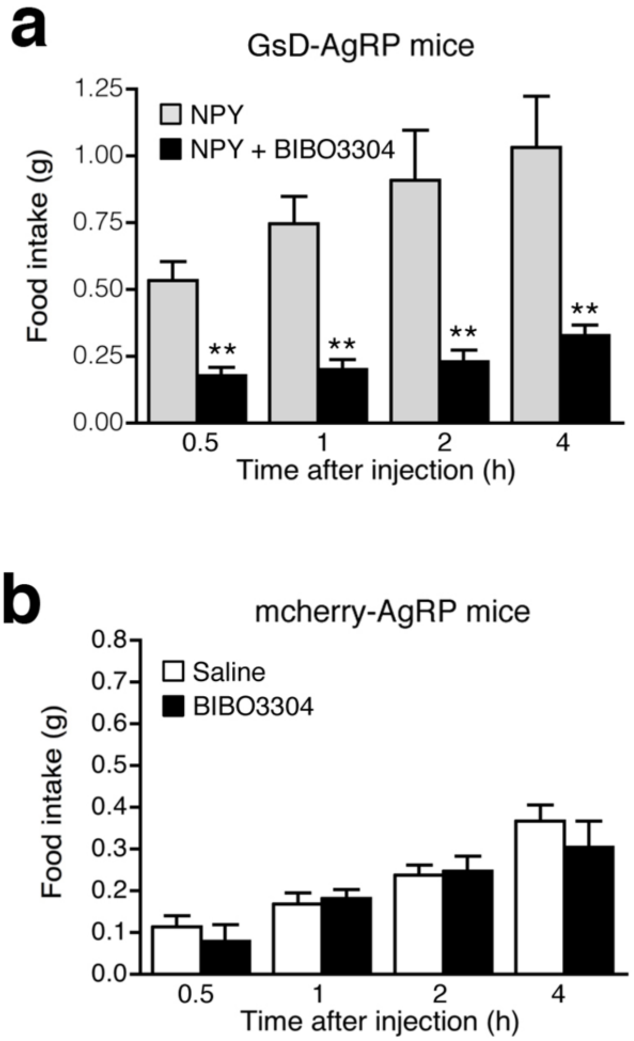


**Supplementary Figure 5.** An NPY $Y_1$ receptor blocker prevents NPY-induced increases in food intake. (**a**) GsD-AgRP mice were injected i.c.v. with NPY (0.85 µg) or NPY (0.85 µg) plus BIBO3304 (3.3 µg), a selective NPY $Y_1$ receptor antagonist (injection time: 9 to 10 a.m.). Food intake was monitored over the indicated period of time (15-week-old males; n=3 or 4 per group). (**b**) Using the same experimental protocol, control mice (mcherry-AgRP mice) were injected i.c.v. with either saline or BIBO3304 (3.3 µg) alone (13-15-week-old males; n=4 or 5 per group). Data are given as means ± s.e.m. **p<0.01 as compared to the group injected with NPY alone (Student's *t*-test).

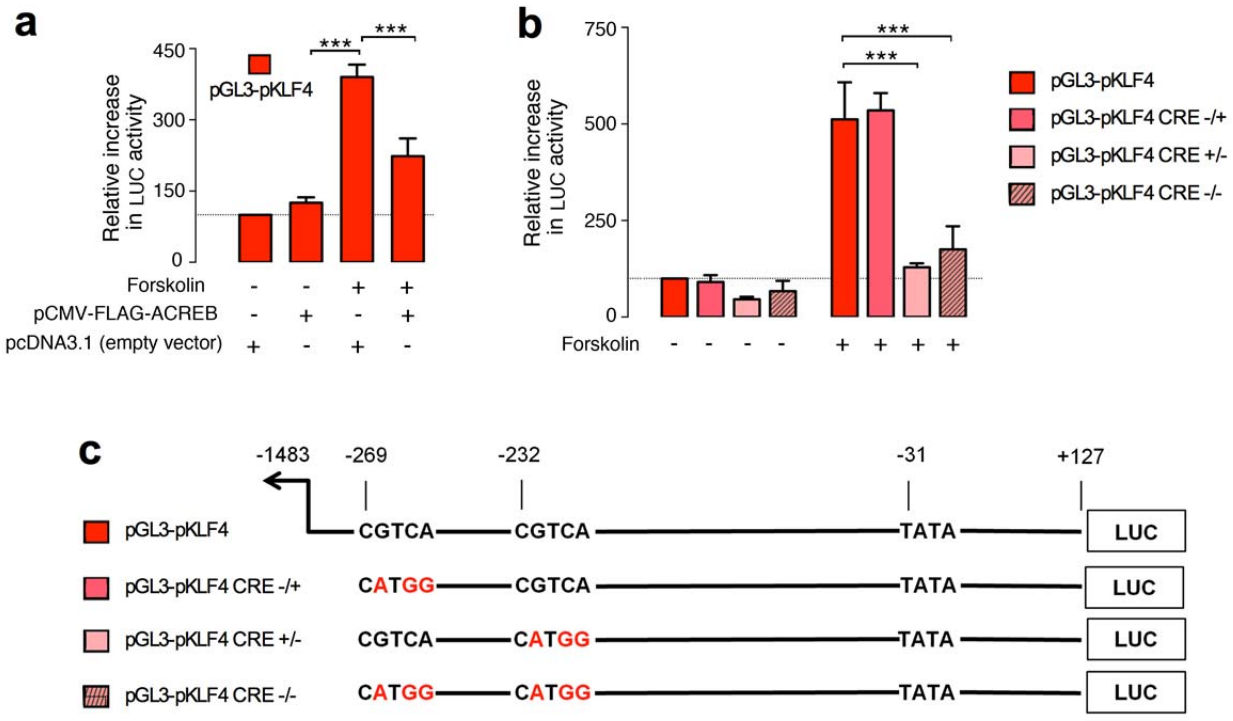


**Supplementary Figure 6.** The *KLF4* promoter is a direct target of activated CREB. (**a, c**) For *KLF4* promoter-luciferase reporter assays, the pGL3-pKLF4 luciferase reporter construct was expressed in HEK293T cells. Following incubation of transfected cells with forskolin (10 µM) or vehicle (DMSO), luciferase (LUC) activity was determined. Forskolin stimulation resulted in a robust increase of LUC activity in the pGL3-pKLF4-transfected cells. This response was greatly reduced after co-transfection of pGL3-pKLF4 with pCMV-FLAG-ACREB which codes for a dominant negative version of CREB. (**b, c**) The two half CRE sites contained within the *KLF4* promoter were mutated by site-directed mutagenesis. Mutation of the second half CRE site (at -232), but not of the first half CRE site (at -269), almost completely abolished forskolin-mediated stimulation of *KLF4* promoter activity. Data are presented as means ± s.e.m. of three independent experiments each performed in duplicate (***$p < 0.001$; two-way ANOVA, Tukey's multiple comparisons test).

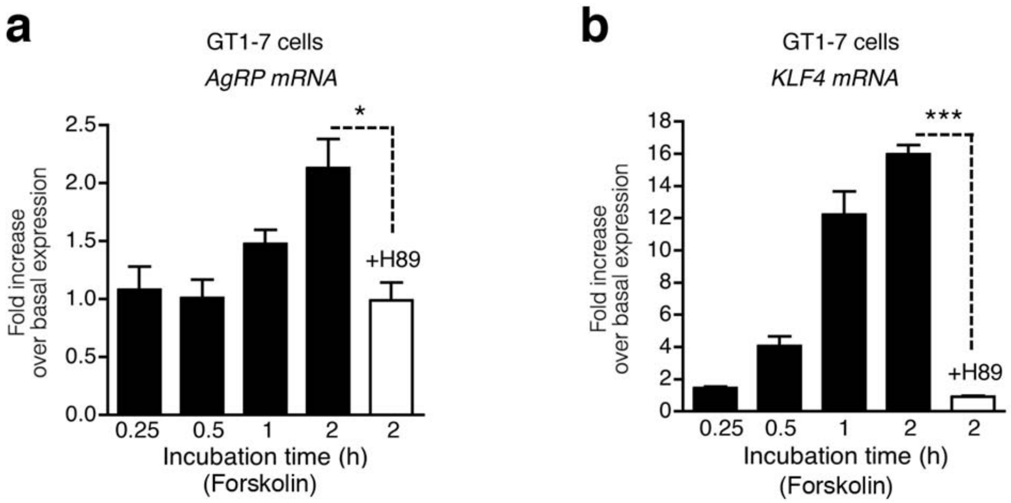


**Supplementary Figure 7.** Effect of forskolin on *AgRP* and *KLF4* mRNA expression in GT1-7 cells. **(a, b**) GT1-7 cells were incubated with forskolin (10 μM) for the indicated periods of time, followed by measurement of *AgRP* (a) and *KLF4* (b) transcript levels. Please note that addition of the PKA inhibitor, H89 (75 μM), completely suppressed the forskolin-induced increases in *AgRP* and *KLF4* expression. Data are given as means ± s.e.m. (n=4). p<0.05*, p<0.001***, as compared to the corresponding H89-treated group (Student's *t*-test).

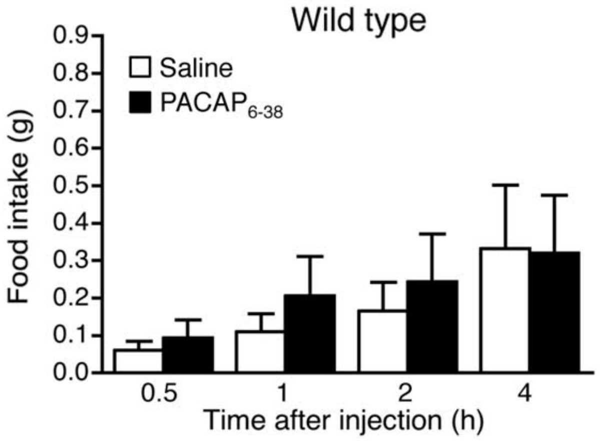


**Supplementary Figure 8.** $PACAP_{6\text{-}38}$ has no significant effect on food intake in WT mice. WT mice (28-week-old males) received a single i.c.v. injection of either saline or $PACAP_{6\text{-}38}$ (1.2 μg), a potent $PAC_1$ receptor antagonist. Food intake was measured after the indicated periods of time. Data are given as means ± s.e.m. (n=4 per group).

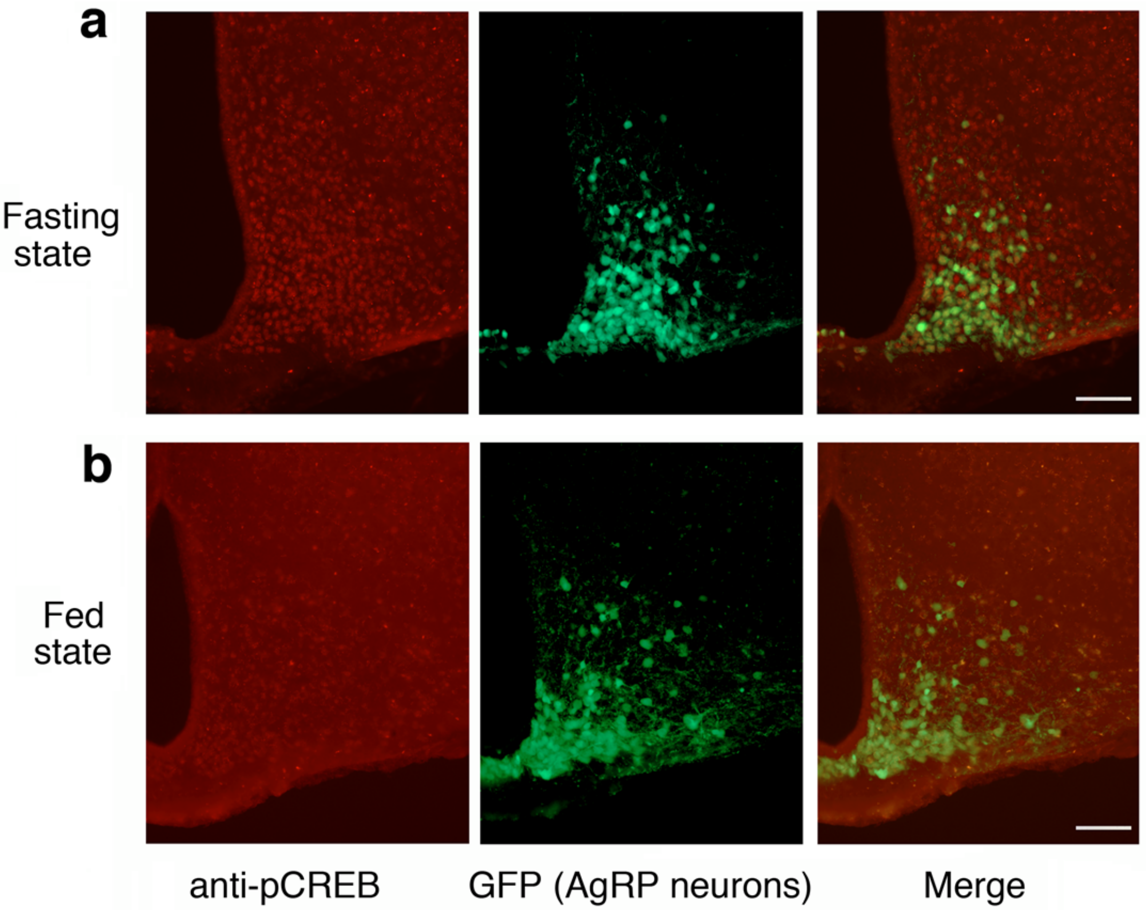


**Supplementary Figure 9.** Fasting-induced increases in pCREB expression in AgRP neurons. This figure shows representative low-magnification confocal images. **(a)** Representative images of hypothalamic sections obtained from *Npy-GFP* mice after a 20 hr fast. pCREB-expressing cells are visualized by an anti-pCREB antibody (red stain, left panel). AgRP (NPY)-positive neurons are visualized by green GFP fluorescence (center panel). The right panel represents a merged image indicating that most AgRP neurons are pCREB-positive. (**b**) Images corresponding to the ones shows in (a) were obtained with freely fed *Npy-GFP* mice, indicating a striking reduction in pCREB-positive AgRP neurons. Scale bars, 100 μm.

**Supplementary Table 1**

Functional expression of the GsD designer receptor containing a C-terminal eGFP tag in COS-7 cells

| DREADD | [$^3$H]NMS binding | | CNO-induced cAMP accumulation | | |
|---|---|---|---|---|---|
| | $K_D$ | $B_{max}$ | $EC_{50}$ | Basal cAMP | $E_{max}$ cAMP |
| | *nM* | *nmol/mg of protein* | *nM* | *pmol/well* | *pmol/well* |
| GsD | 1.5 ± 0.1 | 11.7 ± 1.7 | 4.2 ± 1.4 | 2.2 ± 0.5 | 17. 1 ± 2.4 |
| GsD-eGFP | 2.1 ± 0.2 | 16. 2 ± 2.6 | 4.9 ± 1.6 | 1.1 ± 0.1 | 8.6 ± 1.3 |

[$^3$H]NMS saturation binding studies and cAMP assays were carried out with transiently transfected COS-7 cells, as described by Guettier et al.[16]. Data are given as means ± s.e.m. of three independent experiments carried out in duplicate. No significant [$^3$H]NMS binding or CNO-induced cAMP accumulation was observed with control cells transfected with empty vector DNA.

**Supplementary Table 2**

Summary of antibodies used for immunohistochemical studies

| Antibody target | Source of antibody | Catalog # | Dilution |
|---|---|---|---|
| GFP | Abcam, Cambridge, MA | ab6662 | 1:400 |
| AgRP | Phoenix Pharmaceuticals, Burlingame, CA | H-003-57 | 1:1,000 |
| c-fos | Abcam, Cambridge, MA | ab99515 | 1:500 |
| RFP (mcherry) | Abcam, Cambridge, MA | ab34764 | 1:400 |
| pCREB | Cell Signaling Technology, Danvers, MA | 9198 | 1:500 |
| KLF4 | R&D Systems, Minneapolis, MN | AF3640 | 1:200 |
| HA | Cell Signaling Technology, Danvers, MA | 3724 | 1:500 |
| Anti-rabbit IgG, Alexa Fluor® 594-conjugated secondary antibody | Abcam, Cambridge, MA | ab150064 | 1:1,000 |
| Anti-rabbit IgG, FITC conjugated secondary antibody | Life Technologies, Frederick, MD | A11008 | 1:1,000 |
| Anti-goat IgG, Alexa Fluor® 488-conjugated secondary antibody | Abcam, Cambridge, MA | ab150137 | 1:1,000 |
| Anti-goat IgG, Alexa Fluor® 594-conjugated secondary antibody | Life Technologies, Frederick, MD | A11058 | 1:1000 |

**Supplementary Table 3**

Summary of PCR primers used for qRT-PCR experiments

| **Mouse gene** | **Primer sequence** | **Amplicon (bp)** |
|---|---|---|
| *β-actin* | Forward: 5’ CTAAGGCCAACCGTGAAAAG<br>Reverse: 5’ ACCAGAGGCATACAGGGACA | 104 |
| *AgRP* | Forward: 5’ TTTGTCCTCTGAAGCTGTATGC<br>Reverse: 5’ GCATGAGGTGCCTCCCTA | 86 |
| *KLF4* | Forward: 5’ CACACAGGCGAGAAACCTTACC<br>Reverse: 5’ CGGAGCGGGCGAATTT | 64 |
| *NPY* | Forward: 5’ CCGCTCTGCGACACTACAT<br>Reverse: 5’ TGTCTCAGGGCTGGATCTCT | 68 |
